# A Label-free Mass Spectrometry Method to Predict Endogenous Protein Complex Composition*[Fn FN2]

**DOI:** 10.1074/mcp.RA119.001400

**Published:** 2019-06-11

**Authors:** Zachary McBride, Donglai Chen, Youngwoo Lee, Uma K. Aryal, Jun Xie, Daniel B. Szymanski

**Affiliations:** ‡Department of Botany and Plant Pathology, Purdue University, West Lafayette, Indiana; §Department of Statistics, Purdue University, West Lafayette, Indiana; ¶Purdue Proteomics Facility, Bindley Biosciences Center, Discovery Park, Purdue University, West Lafayette, Indiana; ‖Department of Biological Sciences,Purdue University, West Lafayette, Indiana

**Keywords:** Macromolecular complex analysis, Protein complex analysis, Label-free quantification, Arabidopsis, Plant Biology*, Protein correlation profiling

## Abstract

Information on the composition of protein complexes can accelerate mechanistic analyses of cellular systems. Protein complex composition identifies genes that function together and provides clues about regulation within and between cellular pathways. Cytosolic protein complexes control metabolic flux, signal transduction, protein abundance, and the activities of cytoskeletal and endomembrane systems. It has been estimated that one third of all cytosolic proteins in leaves exist in an oligomeric state, yet the composition of nearly all remain unknown. Subunits of stable protein complexes copurify, and combinations of mass-spectrometry-based protein correlation profiling and bioinformatic analyses have been used to predict protein complex subunits. Because of uncertainty regarding the power or availability of bioinformatic data to inform protein complex predictions across diverse species, it would be highly advantageous to predict composition based on elution profile data alone. Here we describe a mass spectrometry-based protein correlation profiling approach to predict the composition of hundreds of protein complexes based on biochemical data. Extracts were obtained from an intact organ and separated in parallel by size and charge under nondenaturing conditions. More than 1000 proteins with reproducible elution profiles across all replicates were subjected to clustering analyses. The resulting dendrograms were used to predict the composition of known and novel protein complexes, including many that are likely to assemble through self-interaction. An array of validation experiments demonstrated that this new method can drive protein complex discovery, guide hypothesis testing, and enable systems-level analyses of protein complex dynamics in any organism with a sequenced genome.

## Abstract (precis)

At least one third of soluble proteins are predicted to exist in a stable oligomeric state. However, the compositions of the vast majority are unknown. This paper describes a biochemical method to predict protein complex composition based on orthogonal chromatographic separations and label-free protein correlation profiling. The validated method predicts hundreds of novel homo- and heterooligomeric complexes, and provides a new way to analyze protein complexes in any organism with a well-annotated proteome.

## Graphical Abstract

**Figure:**
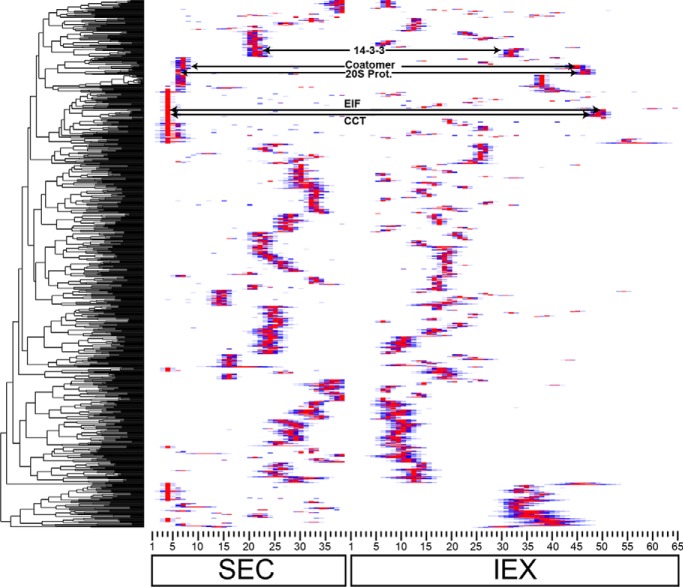

**Highlights**

- Endogenous protein complex composition was predicted using orthogonal protein separations, protein correlation profiling, and novel data filtering scripts.
- The validated method accurately identifies homo- and heterooligomeric complexes.
- Profiling of the *AIMP1* mutant validated the discovery of a t-RNA synthetase-clustering complex.

There are important roles for “omics” technologies to generate systems level data to inform strategies for trait engineering (1, 2). Information about protein oligomerization is some of the most valuable biological data that can provide insight into the control of metabolic pathways and cellular systems (3–5). Protein complex composition identifies genes that function in a common pathway (6). Protein complex formation can also strongly influence metabolism, as oligomerization can control enzyme activity, alter substrate specificity, and define metabolic flux into distinct pathways (4, 7). Protein complex composition provides insight into how molecular machines form vesicles (8) or recognize, unfold, and degrade ubiquitinated proteins (9). Protein complexes also can serve as coincidence detectors to convert multiple input signals into a coherent output (10). Cytosolic proteins also impact the complex shape of a plants cell and organs by regulating cytoskeletal proteins and cell wall properties (11). A single protein can assemble into multiple distinct protein complexes, providing important clues about how distinct cellular pathways might be integrated (12).

Using the plant model Arabidopsis, it is estimated that about one third of the cytosolic proteins exist as a subunit of a stable complex (13); however, the composition of the vast majority remains unknown. This is largely because protein-protein interactions cannot be predicted by genome sequence or expression data alone, and a biochemical experiment is required to detect physical interactions. There are many effective methods to test for protein complex formation in a high throughput manner (13–21). The yeast-two-hybrid assay was adapted to high throughput workflows to detect binary protein-protein interactions (22). Large scale yeast-two-hybrid datasets can be analyzed to indirectly predict higher order protein complex composition by generating networks of interactors; however, the probability of false positives increases as the number of interactors increases (23, 24). Native complexes can be isolated and identified with antibodies and coimmunoprecipitation (CoIP)^1^ or tandem affinity purification coupled with mass spectrometry (25–27). This requires either robust antibodies (28) or the generation of transformed organisms in which the affinity-tagged protein is functional and expressed at appropriate levels to minimize artifactual protein complex formation (14, 22, 29, 30).

Protein correlation profiling is an attractive method to analyze endogenous protein complexes as a function of their elution profiles. Protein complex composition prediction is based on the premise of “guilt by association” in which subunits of stable protein complexes coelute independent of the purification strategy. The method is enabled by the parallel protein quantification inherent to modern protein mass spectrometry and the availability of high-quality proteomes. Increased protein coverage and accurate quantification is being driven by improvements in mass spectrometry instrumentation and data analysis pipelines (13, 21, 31–33).

In practice, protein complex composition prediction is challenging because the cell extract is a mixture of thousands to tens of thousands of monomers and complexes. Using size-based separations it is possible to measure the apparent mass of hundreds to thousands of proteins in a single experiment, and the subset that is likely to exist in an oligomeric state (13, 31, 32, 34, 35). However, chance coelution limits one's ability to accurately predict complex composition based solely on an SEC separation. As an alternative approach, machine learning and bioinformatic algorithms that combine LC/MS profile data with gene coexpression, coevolution, and protein-protein interaction datasets have been used to make more restricted predictions about protein complex composition (36, 37). One way these algorithms are validated is through prediction of known protein complexes. In Arabidopsis, subunits of known, evolutionarily conserved protein complexes rarely exist as stable, fully assembled forms (32). Therefore, mass spectrometry profile data can be incongruent with orthogonal datasets that are constructed based on “golden standards” of assumed fully assembled complexes.

Our goal here is to develop a protein correlation profile workflow in which imperfect but highly useful protein complex composition predictions can be made based on LC/MS profile data alone. Soluble Arabidopsis leaf extracts, enriched in soluble cytosolic and chloroplast proteins, were separated by SEC and IEX chromatography to generate thousands of elution profiles. Automated data filtering of biological replicates was used to identify and combine reproducible profiles and subject them to distance-based clustering analyses to identify groups of proteins with similar elution profiles. The intrinsic information content of the dendrogram and the behaviors of selected known proteins complexes were used to divide the dendrogram and generate specific protein complex composition predictions. An array of biochemical and genetic validation experiments demonstrates the utility of this dataset and the potential use of this method to generate systems-level knowledge about protein complex composition and dynamics.

## MATERIALS AND METHODS

### 

#### 

##### Experimental Design and Statistical Rationale

For LC-MS/MS profiling two biological replicates were used based on the high level of reproducibility between replicates. In previous studies most of the proteins had a reproducible peak between biological replicates (13, 21, 32). Ion exchange chromatography provided a high-resolution separation and 65 fractions were analyzed by mass spectrometry (analyzed on Sciex 5600 mass spectrometer). For the SEC and IEX profiling experiments that were analyzed to predict protein complex composition were analyzed on Sciex 5600 mass spectrometer. The SEC fractions that were analyzed to test for oligomerization changes in predicted AIMP1L-interactors by profiling the *aimpl1* mutant were analyzed on Q Exactive mass spectrometer. For CoIP-MS pull downs three replicates were performed with antibodies against the protein of interest and negative controls and were analyzed on Q Exactive mass spectrometer.

##### Plant Growth and Cell Fractionation

Arabidopsis *thaliana* ecotype Colombia was grown in tissue culture under continuous light (0.5× MS salts, 1% sucrose, 0.8% Bacto agar) for 21 days after germination (13). Two grams of leaf tissue was collected and all the remaining steps were performed immediately without freezing at 4 °C on ice. The leaves were transferred to a 50 ml round bottom centrifuge tube with 7 ml of ice-cold MIB buffer (50 mm HEPES-KOH pH 7.5, 250 mm sorbitol, 50 mm KOAc, 2 mm Mg(OAc)_2_, 1 mm EDTA, 1 mm EGTA, 1 mm DTT, 2 mm phenyl methyl sulfonylfluoride and 1% (v/v) inhibitor mixture (160 mg/ml benzamidine-HCl, 12 mg/ml phenanthroline, 0.1 mg/ml aprotinin, 100 mg/ml leupeptin, and 0.1 mg/ml pepstatin A) for homogenization. Two 10 s bursts of a polytron (Brinkmann Instruments, Riverview, FL) homogenized the tissue. Debris was removed by filtration of the homogenate through four layers of cheesecloth. Differential centrifugation enriched the soluble proteins by spinning at 1000 × *g* (Beckman Avanti 30, Alanta, GA) for 10 min, 4 °C. The supernatant was enriched by pelleting membranes by ultracentrifugation at 200,000 × *g* for 20 min, 4 °C (Beckman Optima Ultracentrifuge). The remaining supernatant contained the crude cytosolic proteins. RuBisCO was depleted from the crude cytosolic fraction using Seppro RuBisCO spin columns according to the manufacturer's specifications (Sigma Aldrich, St. Louis, MO).

##### Size Exclusion and Ion Exchange Chromatography

Size exclusion chromatography was performed on an AKTA FPLC system (GE Life Sciences, Pittsburgh, PA) using either a Superdex increase 200 10/300 GL (GE Healthcare) or HiLoad 16/600 Superdex 200 pg column (GE Life sciences). The mobile phase was [50 mm HEPES-KOH pH 7.8, 100 mm NaCl, 10 mm MgCl_2_, 5% glycerol and 1 mm DTT] and flow rates were 0.6 ml/minute for the 10/300 column and 1 ml/min for the 16/600 column. Protein loading was 0.5 ml (∼1 μg total protein) for the 10/300 and 2 ml (∼4 mg total protein) for the 16/600 column. The columns were calibrated using the gel filtration kit 1000 (MWGF1000, Sigma-Aldrich) using standards ranging from 669 to 29 kDa and the void was determined using blue dextran as previously described (13). Fractions were collected starting at the void to ∼5 kDa.

For separation by charge using ion exchange chromatography a buffer exchange was required for effective protein binding to the solid phase. Buffer exchange was performed using Amicon ultra-15 50 ml centrifugal filters (Milipore, Burlington, MA) to exchange into 20 mm Tris/HCl pH 7.5. IEX chromatography was performed using a Dionex Ultimate 3000 UPLC (Thermo Fisher, Waltham, MA) and a PolyLC (Columbia, MD) mixed bed ion exchange column in Buffer A [20 mm Tris/HCl pH 7.5, 5% glycerol, and 0.5 mm DTT] then eluted with a 35 min linear gradient to increase the mobile phase to 50% buffer A and 50% Buffer B [20 mm Tris/HCl pH 7.5, 5% glycerol, 1.5 m NaCl and 0.5 mm DTT] and over the final 5 min the buffer composition was ramped to 25% Buffer A and 75% Buffer B. Sixty-five 500 μl fractions were collected.

##### Gel Electrophoresis

Proteins were separated by SDS-PAGE and visualized with Coomassie blue staining using standard procedures. Proteins were loaded by equal proportions in 1× Laemmli buffer [0.1 M Tris-HCl, pH 6.8, 1% SDS and 5% glycerol] onto 10% gels and stained with Coomassie blue [50% Methanol, 10% acetic acid and 0.0125% Coomassie blue].

##### LC-MS/MS Sample Preparation and Analysis

For mass spectrometry analysis, proteins were digested to peptides as described in (32). Briefly, the chromatography mobile phase was removed by acetone precipitation, proteins were solubilized and denatured with urea and digested with trypsin. Peptide concentrations were measured with a BCA assay and the most concentrated sample was adjusted to have a peptide concentration of 0.2 μg/μl and an injection volume of 5 μl was analyzed by mass spectrometry.

##### AB Sciex 5600

For the AB Sciex 5600, SEC and IEX samples were analyzed by LC-MS/MS as described by Aryal *et al.* (21). In brief, an Eksigent nano-LC 425 HPLC (Dublin, CA) separated the peptides over a 90 min 0 to 35% acetonitrile gradient. For the AB Sciex 5600, a quadruple time-of-flight mass spectrometer operated in a data-dependent mode.

##### Thermo Fisher Q Exactive

For the Thermo Fisher Q Exactive high field mass spectrometer, samples were analyzed by reverse-phase HPLC-ESI-MS/MS using the Dionex UltiMate 3000 RSLC nano System coupled to the Q Exactive High Field (HF) Hybrid Quadrupole Orbitrap MS (Thermo Fisher Scientific) and a Nano- electrospray Flex ion source (Thermo Fisher Scientific). Peptides were loaded onto a trap column (300 mm x 5 mm) packed with 5 mm 100 Å PepMap C18 medium and washed using a flow rate of 5 μl/minute with 98% purified water/2% acetonitrile (ACN)/0.01% formic acid (FA) for 5 min. Peptides were separated using a reverse phase Acclaim PepMap RSLC C18 analytical column using a 120-min method at a flow rate of 300 nl/minute. The analytical column was packed with 100 Å PepMap C18 medium (Thermo Fisher Scientific). Mobile phase A consisted of 0.01% formic acid in water and a mobile phase B consisted of 0.01% FA in 80% ACN. The peptides were separated over a linear gradient started at 5% B and reached 30% B in 80 min, 45% B in 91 min, before the column was washed and regenerated. The sample was injected into the QE HF through the Nanospray Flex™ Ion Source fitted with an emission tip from Thermo Scientific. Column temperature was maintained at 35 °C. MS data was acquired with a Top 20 data-dependent MS/MS scan method. The full scan MS spectra were collected over 300–1,650 m/z range with a maximum injection time of 100 milliseconds, a resolution of 120,000 at 200 *m*/*z*, spray voltage of 2 and AGC target of 1 × 10^6^. Fragmentation of precursor ions was performed by high-energy C-trap dissociation (HCD) with the normalized collision energy of 27 eV. MS/MS scans were acquired at a resolution of 15,000 at 200 *m*/*z*. The dynamic exclusion was set at 20 s to avoid repeated scanning of identical peptides.

##### Peptide Identification and Quantification

MaxQuant software (v. 1.5.3.28) (38) was used to analyze and align the LC-MS raw data files, with its built-in Andromeda search engine (39). The search was performed with all the fractions in the biological replicates analyzed together in a single search. The MS/MS spectra were searched against the TAIR (The Arabidopsis Information Resource) protein sequence database version 10 (TAIR10; 35386 protein sequences, 14,482,855 residues) for protein identification. A minimal length of six amino acids was required in the database search. The search was performed with the precursor mass tolerance set to 10 ppm and MS/MS fragment ions tolerance was set to 40 ppm. Database search was performed with enzyme specificity for trypsin, allowing up to two missed cleavages. Oxidation of methionine was defined as a variable modification, and carbamidomethylation of cysteine was defined as a fixed modification. The “unique plus razor peptides” were used for peptide quantitation. The false discovery rate (FDR) of peptide and protein identification was set at 1%. Proteins identified by a single spectra were accepted because they were identified in a minimum of two independent experiments.

To increase the number of peptides that can be used for peptide extracted ion chromatogram (XIC)-based quantification and protein quantification and relative abundance profiling across SEC fractions, the “match between runs” function was enabled in a search containing all raw files with a maximum retention time window of 1 min (21). This “match between runs” allows the transfer of peptide identification between precursor ion signals in nearby fractions in the absence of peptide sequencing by MS/MS spectra, utilizing their accurate mass and aligned retention time (38). Protein and peptide groups were exported as .txt files and additional analysis was performed using Matlab, R, Microsoft Access, and Excel.

##### Reproducibility, Peak Fitting, and Clustering Analyses

Proteins with reproducible profiles were determined by the optimized Gaussian fitting algorithm described in (32). To summarize, proteins were selected for Gaussian fitting when they had ≥2 adjacent nonzero fractions. Based on the resolution of the column, up to four Gaussian peaks were allowed; however adjacent peaks had to be separated by a minimum of four fractions. The maximum shift in peak location between biological replicates was selected based on the number of fractions collected and the peak capacity of the column. A Bayesian information criterion was used that added a penalty to each additional fitted peak to reduce over fitting (40). When a protein did not have two adjacent nonzero fractions, the fraction with the highest intensity was used as the peak, and raw intensity values were retained for all fractions. A matrix of all the peaks in the two biological replicates for a protein was used to find the reproducible peaks that were separated by ≤2 fractions for SEC or ≤4 fractions for IEX.

##### Clustering Analysis and Data Filtering

Hierarchical clustering was used to generate groups of proteins with the most similar elution profiles. To reduce noise in the analysis Gaussian fitted peaks were used when available. For proteins not fitted to a Gaussian peak the raw profiles were used. The intensity range of the protein profiles was normalized from zero to one. Clustering analysis was performed on SEC only, IEX only, and concatenated SEC+IEC datasets. The concatenated dataset was comprised of the combined profiles for the subset of proteins that had reproducible peak location across all replicates and both column types. Clustering assigned proteins into groups/individual clusters based on the similarity of their elution profiles. Similarity of protein pairs was measured by the squared Euclidean distance, which is the sum of the squared difference of the pair (41). For the SEC peaks, the first peak was used that corresponds to the peak with the largest M_app_. In rare cases in which multiple peaks were present in the SEC and IEX profiles for the same protein there would be uncertainty regarding the correct correspondence between the SEC and IEX peaks. In these instances, the peak with the largest M_app_ was used because this corresponds to the protein peak that is most likely to participate as a complex subunit. IEX profiles that contained multiple peaks were deconvoluted and split into multiple entries with a sequential suffix based on the peak number. The dendrogram results were made available at a wide range of divisions with different cluster numbers to enable individuals to search for candidate proteins in nearby clusters with similar elution profiles. A specific protein complex composition was generated by analyzing the dendrogram at a cluster number that was designed to have a high cluster number that decreased false positives, but not too high to separate subunits of known complexes and increase false negatives. This was achieved by analyzing the intactness and purity of known complexes and by analyzing the inherent resolution of the combined datasets. To analyze the intrinsic resolution of the data, we calculate the distance within a cluster. A cluster center is first obtained as the average profile of all proteins in the cluster. The distance within the cluster is the average distance of proteins from the cluster center. The average within cluster distance is calculated as a function of increasing cluster number. Orthologs to known metazoan complexes were used as to identify the small subset of known complexes that were likely to be fully assembled (21). The behaviors of known complexes were used to guide the final cluster number for protein complex predictions. Intactness and purity can be used known protein complexes as a standard to evaluate the clustering result (42). Intactness measured the fraction of subunits from a known complex that fell into a single cluster. Intactness was calculated by taking the maximum number of subunits in a single cluster divided by the total number of subunits of the known complex. Purity determined the fraction of proteins in a cluster that were subunits of the known complex. Purity was measured by taking the cluster with the highest number of subunits from a known complex and calculating the fraction of known subunits divided by the total number of proteins in the cluster.

$$Intactness=\frac{Number\;of\;subunits\;from\;a\;complex\;in\;a\;\sin gle\;cluster}{\text{Total number of complex subunits identified}}$$

$$Purity=\frac{Max\;number\;of\;subunits\;from\;a\;complex\;in\;a\;\sin gle\;cluster}{\text{Total number of proteins in the cluster}}$$

##### Coimmunoprecipitation and LC/MS

Coimmunoprecipitation was performed using either a GFP tagged protein or antibodies with known specificity to the protein of interest. For both approaches two grams of leaves were frozen with liquid nitrogen, powdered by grinding in a mortar and pestle, resuspended in 7 ml of MIB buffer, and soluble proteins were enriched with centrifugation (21). Antibodies generated against ACTIN (C4 clone; Millipore) were bound to Pierce protein A/G magnetic beads (Pierce biotechnology, Waltham, Massachusetts). YFP-GAPC2 (43) was pulled down using GFP-Trap (ChromoTek, Hauppauge, NY). The binding reactions were assembled as follows: 350 μl of soluble proteins were brought to a final concentration of 150 mm NaCl, 20 mm HEPES pH 7.2, and 1% NP-40 in 1 ml and incubated overnight at 4 °C on a rocking table. The beads were then washed three times with 20 mm HEPES pH 7.2, 150 mm NaCl, and 1% NP-40 and two times with 20 mm HEPES pH 7.2. The magnetic bead-trapped proteins were eluted from the beads by heating at 65 °C in 8 m urea and prepared for MS analysis as described above. Peptides from these samples were analyzed on a Thermo Fisher Q Exactive as described above.

##### Protein Complex Prediction Validation Using An Arabidopsis Knockout of a Predicted Complex Subunit

To test for an effect of a predicted novel protein complex subunit on the oligomerization state of the protein complex, an independent SEC profiling experiment with biological replicates was conducted using the preparation of soluble proteins from the *aim1pl* (AT2G40660) knock out and wild-type (Col-0). The methods were as described above but were analyzed using the Thermo Q Exactive with improved sensitivity and reproducibility compared with the AB Sciex 5600. The *aimp1* knock out Gabi Kat 220E08 line was confirmed by PCR to contain a T-DNA insertion in exon 2 of AT2G40660 using PCR (44). The elution profiles of the predicted AIMP1L-associated proteins were compared in duplicate samples from wild-type and mutant extracts. As a negative control, all cytosolic tRNA ligases in the Q Exactive dataset were compared.

## RESULTS

### 

#### 

##### A Workflow for Protein Correlation Profiling-based Predictions of Protein Complex Composition

**Fig. 1.**
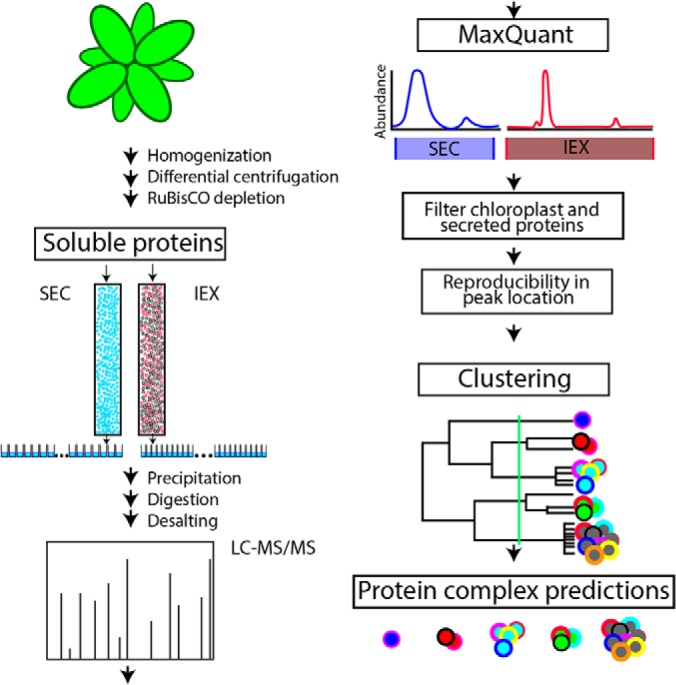
**A label-free proteomic and data analysis workflow to predict the composition of endogenous protein complexes.** Soluble proteins from Arabidopsis shoots were separated under native conditions and in parallel by size exclusion (SEC) and ion exchange chromatography (IEX). Abundance profiles were generated by analyzing each fraction using label-free quantitative mass spectrometry and precursor ion intensities. The method is based on automated peak detection and tests for reproducible peaks in both biological replicates for the SEC and IEX separations. Proteins with reproducible profiles across all separations were subjected to clustering analyses based on the normalized relative protein abundance. Protein complexes were predicted by cutting the resulting dendrogram at a specific location (green line) based on the resolution of the data and benchmarks using known protein complexes.

The objective of this work was to create a label-free proteomic method to predict the composition of endogenous protein complexes from leaf extracts (Fig. 1). An intact organ was used to minimize artifacts caused by generating protoplasts and to facilitate functional analyses of leaves under different growth conditions. Soluble protein extracts were generated from Arabidopsis leaves by homogenization and differential centrifugation. Each biological replicate was split into two samples: half was separated by SEC to obtain an estimate of the apparent mass of the endogenous protein based on its hydrodynamic radius and the other half was separated by charge using a mixed bed IEX column. Proteins in each column fraction were digested and analyzed using quantitative label-free mass spectrometry. Reliable proteomic and bioinformatics data were used to assign the proteins into separate soluble chloroplast and cytosol-enriched datasets (13, 21). Hierarchical clustering analysis was used to group proteins based on the similarity of their elution profiles, and the clustering results were filtered to define a specific cluster number to generate an optimal prediction.

**Fig. 2.**
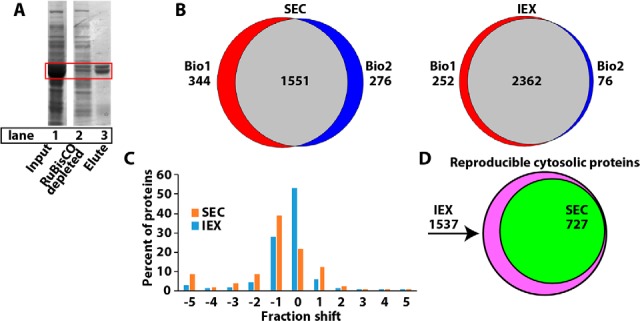
**The workflow generates highly reproducible peaks in both SEC and IEX separations.** *A*, To increase proteome coverage and reduce ion suppression and artifactual peaks, the highly abundant protein RuBisCO was depleted. Lane 1, crude cytosolic input with RuBisCO highlighted in the red box. Lane 2, flow through containing the RuBisCO depleted extract, lane 3, proteins bound to the RuBisCO depletion matrix. *B*, Proteome coverage of identified proteins for SEC (left) and IEX (right) illustrated using Venn diagrams of biological replicates 1 and 2. *C*, Histogram of fraction shifts in peak locations of all proteins that were detected in both biological replicates of the SEC (orange) and IEX (blue) separations. *D*, Overlap in the number of reproducible protein profiles for the SEC and IEX datasets.

Ribulose-1,5-bisphosphate carboxylase/oxygenase (RuBisCO) is a highly abundant chloroplast protein that confounds quantitative proteomics studies because it suppresses the signals of coeluting peptides and caused artifactual peak splitting in profiling experiments (13, 21). In our workflow RuBisCO contamination (Fig. 2*A*, lane 1) was unavoidable because chloroplasts were broken during homogenization. To solve this problem an antibody column was used to deplete RuBisCO to the extent that it was no longer the most prominent protein (Fig. 2*A*, lanes 2 and 3). The RuBisCO-depleted crude cytosolic fraction was separated by SEC and IEX, and fractions were collected for LC-MS/MS profiling. Profiling was performed on two biological replicates of 38 SEC fractions and 65 IEX fractions to identify over 1500 and 2300 proteins in both biological replicates for SEC and IEX, respectively (Fig. 2*B*). There are tradeoffs between sample processing costs, mass spectrometer instrument time, and experimental replication. A previous study relied on a combination of replicates, single runs, and a high number of different separation strategies (36). For this study, we reduced sample processing costs by creating robust chromatography pipelines and automated protein quantification scripts that enabled us to use biological replicates and reproducibility filters to greatly reduce the noise in the data. The raw files have been deposited at JPOST (PXD012601) (45). Supplemental Table S1 contains the raw profiles for proteins and peptides identified in this study. A heatmap of the Pearson correlation coefficients between the biological replicates indicated a high degree of similarity between the biological replicates, with the highest similarity occurring at identical fraction numbers (along the diagonal) for both the SEC and IEX separations (supplemental Fig. S1). The overlap between the SEC and IEX datasets was good with ∼1390 proteins being detected in all four replicates, and this subset was used for further analysis.

Proteins that reside in completely distinct cellular compartments cannot form a protein complex. Although enriched in cytosolic proteins, our sample contains hundreds of chloroplast proteins (13). Chloroplast proteins can be accurately identified based on prior proteomic data, known chloroplast targeting signals, and the sequence of genes encoded by the chloroplast subgenome (13). Therefore, profile data from chloroplast and cytosolic proteins were separated into two groups and separately subjected to a distance-based clustering analysis as described in Aryal *et al.*, 2014. In our dataset 417 chloroplast proteins were subjected to clustering analysis (supplemental Fig. S2*A*). 64 additional proteins were removed because they contained one or more transmembrane domains and appeared to be proteolytic fragments of integral membrane protein, leaving ∼700 cytosolic proteins for the clustering analysis.

In this study, only proteins with a reproducible peak in both replicates were used for protein complex predictions. The use of biological replicates is justified because SEC MS profiling was shown to be highly reproducible in previous studies (13, 21), and similar data filtering procedures have been used here to extract the high-quality reproducible data from the IEX profiles. Peaks in the elution profiles were identified in an automated manner using an optimized Gaussian-fitting algorithm has been previously published and is available as supplemental data (21). To evaluate the fitting error of the Gaussian fitted peaks against the raw data a boxplot of the root mean squared error was plotted for both SEC and IEX replicates. The peak fitting strongly reflected the raw data because the mean of the RMSE is near 0 (supplemental Fig. S2*B*). The R^2^ analyses of the fitting outcomes, which can be interpreted as the square of the correlation between the observed values and the fitted values are displayed in a boxplot, and again a strong correlation was observed with the mean of the boxplot being near 1.0 (supplemental Fig. S2*C*).

The Gaussian-fitting algorithm fitted 81% (591 Gaussian fitted profiles/727 total profiles) of the proteins in the SEC profiling experiment and 97% (705 Gaussian fitted profiles/727 total profiles) in the IEX profiling experiment. In this study, the peak locations in the SEC and IEX separations were reproducible between replicates because 84% of proteins profiled in the SEC had ≤ 2 column fraction shift and 94% in the IEX had ≤ 4 fraction shift (Fig. 2*C*). Using these reproducibility data filters, 727 cytosolic and 402 chloroplast protein profiles were reproducibly measured across all separations (Fig. 2*D*, supplemental Table S2), and this subset was used for protein complex composition predictions.

Not all proteins had a single peak in the SEC and IEX separations, and we did not want to ignore the plausible and biologically relevant possibility that a protein could have combinations of physical associations with itself (homo-oligomerization) and/or unrelated proteins (hetero-oligomerization). The deconvolution of complex elution profiles into individual peaks enabled a protein to have multiple oligomerization states and multiple protein complex predictions (31). In this data set multiple peaks were relatively rare, 86% of the proteins had a single peak in both the SEC and IEX separations (supplemental Fig. S2*D*). Fourteen proteins had multiple peaks on the SEC column. Seventy-six proteins had multiple peaks only on the IEX. This higher number for the IEX likely reflects both the increased resolution of the column and/or the possibility that less stable complexes could partially disassemble during the high salt elution. However, peak locations among 70 cytosolic proteins with multiple IEX peaks were not correlated with high salt concentration, because only 24 of 140 peaks resided in the last third of the column fractions containing the highest salt concentration.

Four proteins had multiple peaks in both the SEC and IEX, making it impossible to accurately pair the deconvoluted SEC and IEX peaks in a clustering-based composition prediction. For these 4 we selected only the one SEC peak with the largest apparent mass, duplicated it, and concatenated these profiles with the deconvoluted peaks from IEX column. In most cases the secondary SEC peak corresponded to the expected mass of the monomeric form making it less important for protein complex prediction anyway. Each of the proteins with multiple peaks in the IEX were given multiple entries (multiple data rows in the profile database) and labeled with a unique “_peak number” suffix. In this way proteins with multiple peaks could be clustered into multiple protein complexes.

##### Evaluation and Optimization of Protein Profile Clustering: Known Complexes and Intrinsic Features of the Dendrogram

**Fig. 3.**
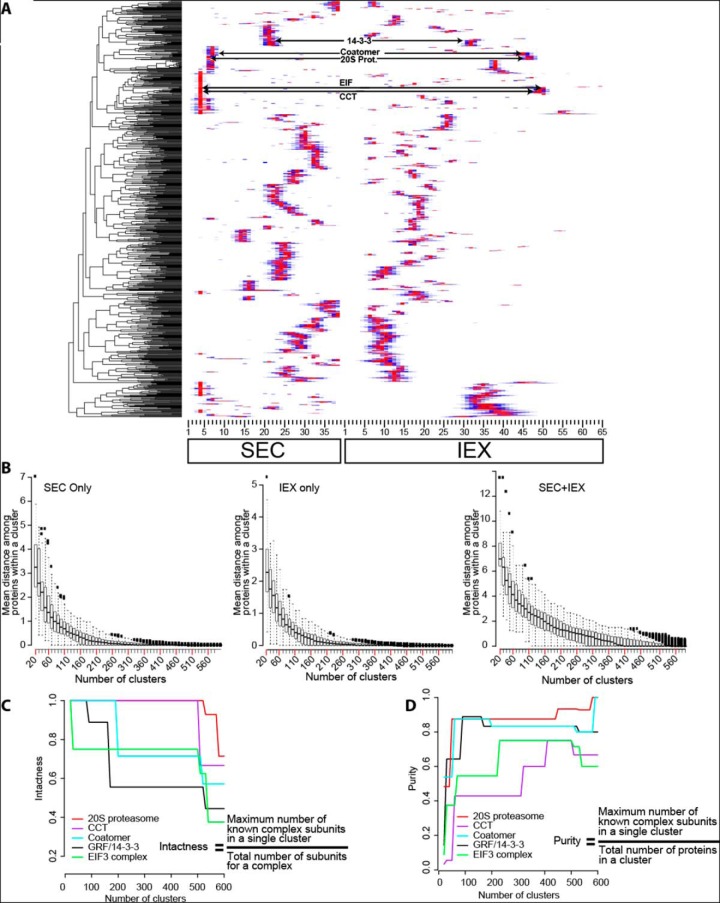
**An example clustering result for a combined pair of SEC and IEX profile data from a split sample and one biological replicate, and methods used to define the location where the dendrogram is divided to make a protein complex composition prediction.** *A*, A hierarchical clustering analysis was performed on the concatenated abundance profile dataset from a single SEC and IEX replicate grouped proteins with similar elution profiles. Subunits of known protein complexes (20S proteasome, CCT, Coatomer, 14–3-3 and EIF3) coeluted. See supplemental Table S2 column D for the subunits of known protein complexes. The arrows point to the precise region of the heatmap where the known subunits eluted. *B*, An intrinsic test of the resolving power of individual and combined profile datasets. Boxplots for the mean distance of protein profile data within the clusters was plotted as a function of increasing cluster number. The distance was defined as the pairwise Euclidean distance of each proteins in the cluster to the mean distance within the cluster. The boxplot represents the first and third quartile of the data with whiskers at 1.5 times the interquartile range. *C* and *D*, Extrinsic tests to guide dendrogram splitting and protein complex composition prediction. *C*, A test to quantify how known protein complexes remain assembled as the dendrogram was divided into an increasing number of clusters. The intactness of the complexes was calculated (see equation in inset) as a function of increasing cluster number. *D*, A test to determine if there were optimal cluster numbers to generate pure clusters containing only the subunits of known protein complexes. The purity (see equation in the inset) of each known complex was calculated as a function of increasing cluster number. The known complexes are color coded as shown in the inset.

**Fig. 4.**
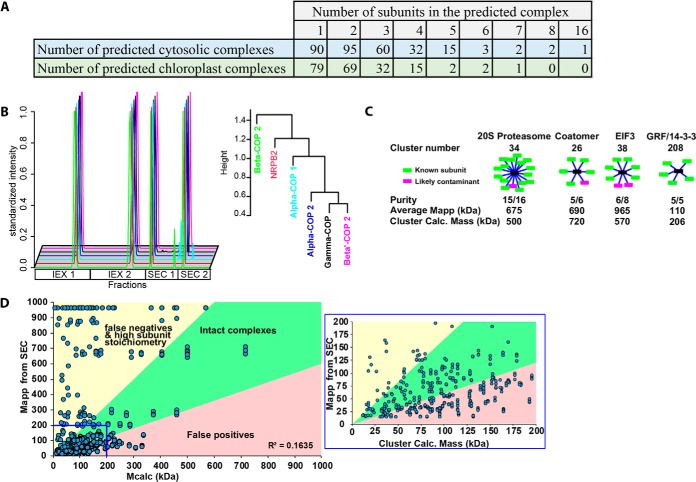
**Protein complex predictions and their evaluation using known complexes and global analyses of predicted complex mass and measured apparent mass from SEC profile data.** *A*, 216 cytosolic protein complexes were predicted that contained two or more subunits. The cartoon shows the subunits as red boxes connected to the black node reflected a group of proteins that may be are associated with each other but do not necessarily directly physically interact. *B*, The SEC and IEX elution profile for each protein in the cluster 26 that contains multiple subunits of the coatomer complex (left, Locus ID in supplemental Table S2 column E). The profiles and dendrogram for cluster 26 were plotted with the known subunits color coded and a single likely contaminant is shown in red (right). *C*, Clusters containing the known complexes, the 20S proteasome, coatomer, EIF3, and 14–3-3 proteins are summarized. The green boxes represent the subunits of known complexes and magenta boxes are either contaminants or new interactors. The purity is shown as the fraction of knowns to total proteins in the cluster. To test if the complex eluted as fully assembled the average M_app_ of the proteins in the cluster was compared with the calculated mass of the predicted complex. The calculated mass of the cluster was determined by the summation of monomer masses for all the proteins in the cluster. *D*, A global comparison of the cluster-based predicted mass of a protein complex (M_calc_) and the measured apparent mass of individual proteins using SEC (M_app_). M_calc_ (*x* axis) is the sum of all the protein masses contained within a single cluster assuming a 1:1 stoichiometry. The plot was divided into three quadrants with the green sector in the middle containing the most reliable predictions because there is less than a 2-fold difference between the M_calc_ and M_app_. The yellow region contains proteins with predicted high subunit stoichiometry with M_app_ 2-fold greater than the cluster calculated mass. This sector may also contain false negatives in which binding partners are either mis-categorized or not deteced. The pink-shaded region contains putative false positives where M_calc_ is more than 2-fold greater than M_app_.

**Fig. 5.**
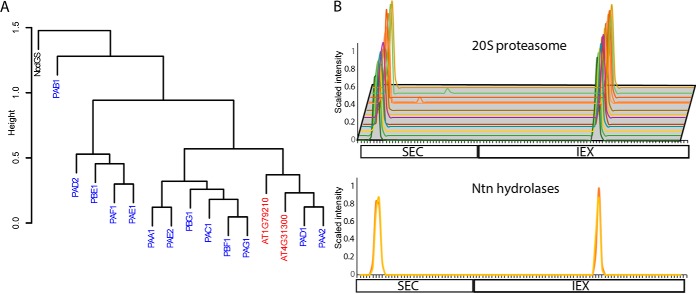
**Validation of protein complex predictions: identification of unannotated 20S proteasome subunits.** *A*, The dendrogram from cluster 32 that contains the 20S proteasome. The names of the 20S proteasome subunits are colored in blue, unannotated Ntn hydrolases in red, and the single likely contaminant in black. *B*, The Gaussian fitted and normalized SEC and IEX abundance profiles for known 20S proteasome (top) and the unannotated Ntn hydrolases (bottom) that were in cluster 32.

Protein complex predictions assume that stable subunits of a protein complex will coelute under any chromatography condition. The heatmap in Fig. 3*A* is an example clustering result using only one biological replicate. The red color represents column fractions with maximal relative protein abundance which were normalized from 0 to 1 allowing proteins to cluster independent of their abundance. Well-resolved protein peaks were distributed across the SEC and IEX column fractions. One highly useful way to validate the clustering result is to test for the coelution of known protein complex subunits. For conserved known protein complexes, the major subunit pool does not exist in a fully assembled state in this profiling workflow (21). Nonetheless, a database of conserved Arabidopsis complexes (32) was mined to identify some useful knowns that could be used to evaluate our predictions. The elution profiles of subunits of the 20S proteasome, heterodimers of 14–3-3/General Regulatory Factors (GRFs), the coatomer vesicle coat complex, the translation initiation factor 3 (EIF3) complex, and chaperonin containing TCP1 folding complex (CCT) complexes coeluted (Fig. 3*A*). Subunits of the coatomer complex (Fig. 4*B*) and 20S proteasome core particle (Fig. 5*B*) coeluted on both columns. The value of the orthogonal IEX separation to resolve complexes that coeluted in the SEC was clear. The coatamer and 20S proteasome complexes coeluted on the SEC, but were clearly resolved on the IEX column (supplemental Fig. S3*A*). Similar increased resolution was evident for the EIF3 and CCT complexes (supplemental Fig. S3*B*). Coelution of multiple subunits from a known complex indicates that all steps of our workflow were reliable because errors in protein identification, quantification, or clustering analysis would generate scattered elution profiles for known complex subunits.

A variety of metrics were developed to analyze the resolution of our datasets and choose a cluster number to divide the dendrogram and generate a specific set of protein complex predictions. One way to analyze the intrinsic resolving power of a clustering result is to calculate the average within cluster distance (described in the previous section) as a function of increasing cluster number. When the average distance within a cluster is high, the elution profiles of the proteins are not as similar compared with when the distance is small. A boxplot showing the mean, first, and third quartiles of the within cluster distance for all clusters in the cytosolic protein dataset was constructed as a function of increasing cluster number (Fig. 3*B*). The plots showed the average distance within clusters was high when the dendrogram was divided into 20 clusters and decreased to zero with increasing cluster number. The within cluster distance approached zero because many of the clusters contained a single protein, meaning there was no distance measured with that cluster. In the boxplot for SEC and IEX alone, the minimum value of the third quartile approached zero at ∼180 clusters (Fig. 3*B*). Concatenating the SEC+IEX profile data from both replicates increased the resolution and the lowest values in the third quartile did not reach zero until ∼300 clusters (Fig. 3*B*), suggesting that cutting the dendrogram of the concatenated IEX and SEC datasets at or near 300 clusters would capture most of the resolving power of the profile data.

Additional metrics were developed to analyze the “intactness” and “purity” of known protein complexes as a function of increasing cluster number (42). Intactness measured the extent to which a complex remained assembled at increasing cluster number by calculating the ratio of the number of subunits in the same cluster to the total number of subunits identified (Fig. 3*C*). All complexes are expected to be grouped within a cluster at low cluster number, but as cluster number increases too far, an increasing number of subunits would be expected to fall into nearby clusters, reducing the “intactness” of the prediction. As expected, the concatenated SEC and IEX profiles outperformed SEC and IEX alone because the 20S proteasome, coatomer, and EIF3 stayed assembled at a higher number of clusters (supplemental Fig. S3*C*). The CCT complex was the exception because it remained fully assembled beyond 600 clusters in SEC only and intactness drops at ∼480 clusters in SEC+IEX. This reflects the distinct behaviors of knowns.

In the clustering result obtained with the concatenated datasets, the intactness and purity responses of the known complexes were variable. The CCT complex and 20S proteasome were extremely stable and had an intactness of one until the dendrogram was cut into >500 clusters (Fig. 3*C*). The GRF/14–3-3/GRF subunits had a sharp decrease intactness near a cluster number of 170. However, this did not necessarily reflect disassembly of GRF subunit containing complexes, because several of the GRFs had multiple peaks on the IEX column, and a small subset of GRFs (GRF1,6,9,8) coeluted at a distinct peak location and clustered as a distinct population of putative heteromeric GRF complexes (supplemental Fig. S4*A* and S4*B*). These distinct predicted GRF complexes may have distinct charge distributions or stabilities on the IEX column that allow them to be cleanly separated. EIF3 and the coatomer also suffered hits to their intactness at increasing cluster number, indicating that in many cases the final predictions will have some false negatives because of a subset of subunits either having a reduced stability in the complex or a more variable elution profile compared with other subunits that remained together at higher cluster numbers.

A purity metric was developed to analyze the effect of increasing cluster number on the frequency of false positive prediction. Complexes are intact but false positive are high at low cluster numbers. The purity increased greatly from 20 to 200 clusters for all complexes (Fig. 3*D*), but for most of the known complexes perfect purity could not be attained, pointing to the unavoidable problem of chance coelution and false positives in our prediction. The intactness and purity indexes suggest the dendrogram be cut between 200 and 500 clusters (Fig. 3*C* and 3*D*). Considering all of the above, the dendrogram from cytosolic protein data was cut into 300 clusters (termed cytosol_300_) and supplemental Table S2 (cytosolic tab) provides the protein composition of each cluster. A cluster number of 300 is a somewhat arbitrary selection. To enable readers to scan the cytosol clustering more broadly for candidate interactors, supplemental Table S3 provides the protein cluster assignment for dendrograms at cluster numbers ranging from 20 and 600 at 10 cluster increments.

The same clustering and data filtering methods described above were used for the chloroplast localized proteins. The chloroplast had ∼400 proteins, and 200 clusters (chloroplast_200_) was chosen because in the distance boxplot the mean is flat and third quartile approaches zero at ∼170 clusters (supplemental Fig. S3*D*). The clustering result for the chloroplast proteins was useful because the large and small subunits of RuBisCO, which are known to physically interact (46), resided within single clusters with perfect purity. Importantly, the behaviors of the large and small subunit of RuBisCO validated our method to analyze proteins with multiple peaks. Both peaks from the large and small subunits clustered together in two different clusters containing only these two known subunits. The composition of the predicted chloroplast_200_ complexes are in supplemental Table S2, chloroplast tab.

To estimate the false discovery rate a clustering analysis was performed on the SEC+ IEX profiles using the both the cytosolic and chloroplast data sets. The assumption is that chloroplast and cytosolic proteins do not physically interact because of distinct compartmentalization, and any cluster that contains a mixture of cytosolic and chloroplast proteins would contain at least one false positive. The percent of clusters containing a mixture of cytosolic and chloroplast proteins was calculated at a range of cluster numbers (supplemental Table S4). At a cluster number of 500, which reflects the resolution used for the individual cytosolic and chloroplast datasets, ∼80% of the clusters were pure containing only cytosolic or chloroplast proteins. This provides an estimate for the false discovery rate for chance coelution.

##### Protein Complex Composition Predictions Based on Coelution

The analyses above generated a specific prediction for 300 cytosolic and 200 chloroplast-localized complexes. At 300 clusters the number of cytosolic proteins in a cluster ranged from 16 (1 instance) to 1 (90 instances) (Fig. 4*A*, supplemental Table S2 column D). There were examples in which subunits of known complexes were highly enriched within a single cluster. For example, coatomer is an heterooligomic protein complex that associates with organelle membranes to promote cargo selection and vesicle trafficking (8). Coatomer contains seven subunits and we detected 5 of them here. Orthologs of the alpha-, beta-, and gamma-subunits showed a high degree of coelution in SEC and IEX separations and segregated together into cluster 26. The epsilon and delta (one of its peaks) fell within the nearby cluster 25 due in part to differences in apparent mass (supplemental Table S2). The EIF3 complex recruits the mRNA to the 40 S ribosome, and is required for translation initiation (25, 47) and 6 of its subunits were grouped into cluster 38 and three other subunits clustered into the nearby clusters 39, 40, and 41 (Fig. 4*C*, supplemental Table S2). The 20S proteasome is a large complex that degrades proteins (9). This study identified 13 known subunits that all clustered together (Fig. 4*C*). The GRF proteins are signaling proteins that can bind to phosphorylated effector proteins or form mixed hetero- and homo-dimers, depending on their subcellular localization (32, 48). Five different GRF isoforms were placed into cluster 208, 3 isoforms in cluster 207, 1 into cluster 206 and 3 isoforms were placed into clusters 8 and 9 (Fig. 4*C*; supplemental Table S2).

Clustering the chloroplast-localized proteins produced 121 clusters containing two or more proteins. Most of the chloroplast clusters had from 2 to 4 proteins. Five clusters contained 5 or more proteins. The known heteromeric RuBisCO complex, which is responsible for CO_2_ fixation during photosynthesis (49) was correctly identified (Supplemental Table 2). The chloroplast-encoded large subunit of RuBisCO and multiple nuclear-encoded RuBisCo small subunits had multiple peaks and each was given 2 protein profile entries. The large and small subunits were assigned to two distinct clusters (clusters 109 and 172) containing only these proteins. In plants thioredoxins are known redox regulators (50) and 18 were found in 14 different putative complexes, pointing to distinct binding partners among the thioredoxins.

We wanted to determine if existing large-scale datasets on gene coregulation or protein-protein interactions were consistent with our chromatography data and had potential use to refine our protein complex predictions. First subunits of known protein complexes were tested for coexpression across a wide array of microarray and RNAseq experiments that were conducted using Arabidopsis (51) and human samples (52). The list of conserved protein complexes common to both Arabidopsis and humans was published previously (32), and pairwise correlation coefficients were used to identify the percent of protein complex subunits that were coexpressed. Although there were a few examples in which all subunits of a complex were coexpressed, the vast majority were not (supplemental Fig. S5*A*). Along similar lines, among the 19 nonself-interacting pairs of Arabidopsis proteins that were in the Biogrid database (53) and in our cytosol dataset, there was not a strong tendency of the protein pairs to coelute on the SEC or IEX columns (supplemental Fig. S5*B*). The low degree of overlap and Biogrid data is somewhat expected because it is derived using methods that often detect a different array of physical interactors compared with those found using affinity-based capture (54, 55). Nonetheless we used the Biogrid data to test for correlations among these previously reported interactions and those predicted from our clustering analysis. Among the 19 protein pairs in Biogrid, 9 had very similar cluster IDs (cluster IDs that had a difference of less than or equal to 2, supplemental Table S5). This level of similarity is not due to chance. When cluster IDs were randomly drawn for the predicted interactors zero pairs were matched in over 70% of the simulations (*n* = 10,000 simulations), and we never observed more than 4 matched pairs in any of the simulations. These analyses indicate that there is significant agreement between our clustering predictions and the Biogrid database.

The results sections below include a wide array of validation studies that demonstrate the utility of these predictions. However, we want to emphasize that this protein complex prediction method is imperfect and contains many false positives and false negatives. Selecting a cluster number of 300 for the cytosol is somewhat arbitrary and differentially affects the purity and intactness of protein complexes (Fig. 3*C* and 3*D*). Parameters like chance coelution of unrelated proteins and complex disassembly during purification contribute to false positives and false negatives, respectively. If the method was perfect and if all subunit stoichiometries were 1:1 (ignores the frequent case of high subunit stoichiometries, see below), then one would expect the summed monomeric masses of all proteins in a cluster (M_calc_) to equal the apparent mass (M_app_) of the protein measured using SEC. The plot of M_app_ *versus* M_calc_ revealed an overall weak correlation between these two measurements (Fig. 4*D*), and only about ∼25% of the complexes fell near the diagonal with less than a 40% difference between the M_app_ and M_calc_.

Therefore, to facilitate judicious use of the prediction results we developed a simple classification scheme to categorize the reliability of the cytosol_300_ and chloroplast_200_ predictions. Clusters and individual proteins were divided into defined classes based on the number of proteins in the cluster, the summed mass of proteins in the cluster (M_calc_), and the measured apparent masses of the individual proteins (M_app_). See supplemental Table S2, column M, for the classification scheme and the category definitions. For example, single protein clusters were defined as “degraded” when the R_app_ (R_app_ = M_app_/M_monomer_) < 0.5 or “monomeric” when 0.5 ≤ R_app_ ≤ 1.6. Homo-oligomerization is commonly evolutionary phenomenon (4), and some solo proteins had a very high M_app_ compared with the cluster M_calc_. Many known homooligomers were present in the upper left sector of Fig. 4D (see also below). Thus, solo proteins were classified as “homooligomer” if the protein had an R_app_ ≥ 1.6. To predict putative homooliogomers or proteins with a high subunit stoichiometry in clusters that might contain a small number of false positives, proteins in clusters with 2 or 3 members that had an M_app_ ≥ (4* M_calc_) were classified as “possible homo- or hetero-oligomer/high subunit stoichiometry”. About 30% of the cytosol_300_ clusters and 35% of the chloroplast_200_ clusters fell into the homomer/heteromer/high subunit stoichiometry categories.

Another likely reliable prediction class had M_calc_ values that were similar to M_app_ of the proteins in the cluster. If a cluster had 2 or more proteins and M_calc_ was within 40% of the average M_app_ (M_app-avg_) of the cluster, then proteins in the cluster were classified as “putative intact complex.” 15% of the cytosol_300_ and 12% of chloroplast_200_ clusters fell into this category. Proteins in this cluster type could also correspond to unstable subunits of large complexes that disassociate during purification on the IEX. There were examples in which a subset of proteins of known complexes had multiple peaks in the IEX and were clustered into a second small cluster compared with subunits of the intact complex. These proteins tended to reside in the upper left sector of the graph because all multiple peak proteins were referenced to a single maximal SEC peak (as explained above). Any protein for which a subset of the IEX peaks fell in the upper left quadrant (M_app_ ≥ (4* M_calc_)) was classified as “subcomplex or high subunit stoichiometry.” Multiple peak proteins in which all peaks had an M_app_ > 1.4* M_calc_ were classified as “partial complex/false negatives”.

False positives because of chance coelution are the most common source of errors in our predictions, and the least reliable clusters fell in the extreme lower right sector of Fig. 4*D*. A cluster containing two or more proteins was flagged for false positives if M_calc_ > 1.4 * M_app_. The R_app_ of individual protein in this cluster type was used to distinguish putative complex subunits from the false positive. If a protein within this cluster type had an R_app_ ≥ 1.6, it was classified as “putative complex clustered with false positives”. Predicted complexes with the highest M_calc_ to M_app_ ratios are the least reliable among our cytosol_300_ and chloroplast_200_ predictions. A protein within this cluster type was flagged as “likely false positive: monomer” if it was expected to be monomeric (R_app_ < 1.6) (*e.g.* see proteins in cluster 107 of the cytosol_300_ prediction). Fourteen percent of the cytosol_300_ and 19% of chloroplast_200_ clusters fell into this least reliable category in which the cluster was comprised entirely of predicted monomeric proteins. These metrics can serve as benchmarks for future complex prediction studies.

##### Validation of Protein Complex Predictions: Unannotated Proteasome Subunits

Our dataset appears to contain useful predictions for hundreds of unannotated proteins and novel complexes. For example, our analysis of the 20S proteasome showed that known subunits of the 20S proteasome formed a nearly pure cluster. The 20S proteasome falls into cluster 34 and contains 16 proteins with 13 being known subunits (Fig. 5*A*) (9, 56). One protein NODGS, had a profile that was most dissimilar to the proteasome subunits and was a likely contaminant. The two additional proteins AT1G7920 and AT4G31300 were N-terminal (Ntn) hydrolases which are known proteases. The SEC and IEX profiles showed that the 20S protease and Ntn hydrolase were nearly identical, and both proteins fell in the middle of the cluster surrounded by known subunits. (Fig. 5*A* and 5*B*). The coelution and homology to known subunits provides strong evidence that these two Ntn hydrolases were indeed unannotated proteasome subunits showing that 15 of the 16 proteins in cluster 34 belong to the proteasome.

##### Validation of Protein Complex Predictions: Known Homooligomers

There was an interesting class of oligomerization predictions in which M_app_ greatly exceeded M_calc_. These behaviors are expected for homooligomers and heterooligomers with high subunit stoichiometries. This sector of the graph is populated by several known homomers. Examples include PYRIDINE BIOSYNTHESIS 1.1 (57–59), Aldolase, (60), Glutamine Synthetase (7). NAP1, which has numerous functions related to histone complex assembly (61), including shuttling newly synthesized histone complexes into the nucleus (62). NAP1 homodimers can assemble into multimeric complexes including hexamers (63), and similarly sized NAP1 complexes are consistently identified in Arabidopsis leaf extracts (13, 21). In the cytosol_300_ prediction here, NAP1 was flagged as a predicted homooligomeric hexamer (supplemental Table S2, column M). This cluster class included phosphofructokinase (PFK) an important enzyme in central carbon metabolism that promotes carbon flux into the glycolytic pathway. The vertebrate PFKL isoform forms filaments of stacked tetramers that are easily resolved by EM and cluster into distinct puncta in living cells (64). Our data predict that PHOSPHOFRUCTOKINASE7 forms homooligomers containing 18 subunits. Additional protein complexes flagged as homooligomer or stoichiometry not 1:1 included: Glutamate decarboxylase a reported hexamer (65), the reported tetramers S-adenosylmethionine synthase (66), Aldehyde dehydrogenase (67) and carbonic anhydrase a known homooctomer (68, 69). The known Arabidopsis hexamer CDC48 was also correctly flagged (70, 71).

##### Validation of a Predicted tRNA Ligase Clustering Complex

**Fig. 6.**
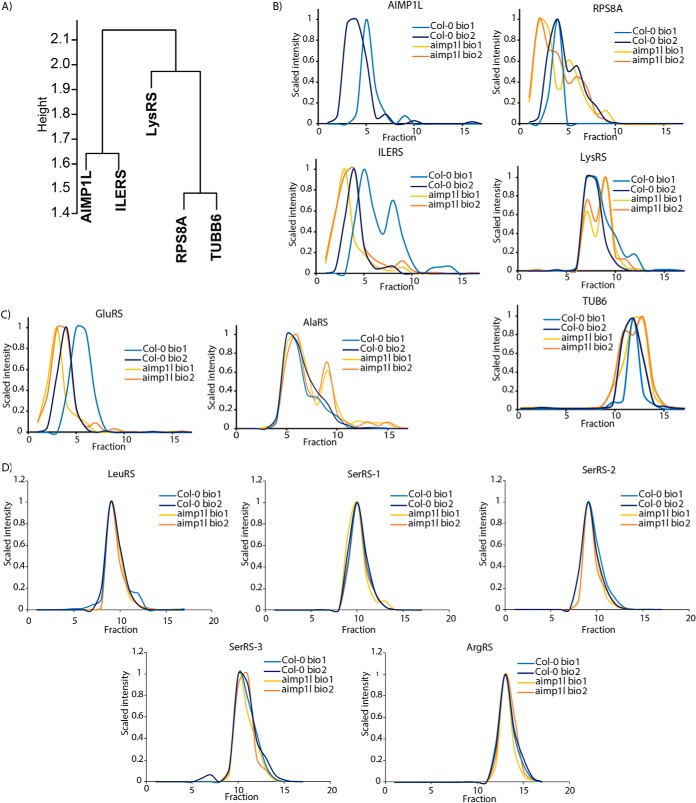
**Validation of a novel cytosolic tRNA ligase clustering complex in Arabidopsis.** Effects of *aimp1l* on predicted protein complex subunits based on the cytosol_300_ clustering result. *A*, AIMP1L is predicted to be a subunit of a novel cytosolic tRNA ligase complex based on the composition of cluster 64. *B*, The raw elution profiles for predicted interactors of AIMP1L. AIMP1L was detected in wild type plants and not detected protein in *aimp1l* (GABI-kat-220E08, AT2G40660) extracts (upper left). Profiles of putative AIM1L-complex subunits in wild-type (light and dark blue profiles) and *aimp1l* (yellow and orange profiles): Ribosomal protein S8A (RPS8A, AT5G20290), Isoleucine-tRNA synthetase/ligase (ILERS, AT4G10320), Lysine-tRNA synthetase/ligase (LysRS, AT3G11710) (bottom right), and tubulin Beta-6 (TUB6, AT5G12250). *C*, Additional tRNA synthetases with altered profiles that were reproducibly detected only in the *aimp1* profile experiment: Glutamyl-tRNA synthetase (GluRS, AT5G26710). And Alanine tRNA synthetase (AlaRS, AT5G22800). *D*, The remaining tRNA synthetases that were detected in the aimp1l profiling experiment but did not have an altered elution profile in the mutant: Leucine tRNA synthetase (LeuRS, AT1G09620), three Serine tRNA synthetase (SerRS, AT1G11870, AT5G27470 and AT5G6680), and Arginine tRNA synthetase (AT4G26300).

To further validate the method, we conducted an open-ended profiling experiment in a mutant background in which a predicted subunit of a novel complex was disrupted by an insertion mutation. In the ideal case, loss of the subunit would cause catastrophic complex disassembly (72) or destabilization of individual subunits in the absence of the assembled complex (73). Alternatively, if the deleted subunits are peripheral, and of sufficient size to significantly affect radius of the partially assembled complex, a shift in the apparent mass could be detected in an SEC profiling experiment (72, 74). In these scenarios, one would expect true positive interactors to coelute in the wild type and have an altered oligomerization state in the mutant. We focused on NUCLEIC ACID-BINDING, OB-FOLD-LIKE PROTEIN/AT2G40660 because it was a single-copy gene, predicted to be in a complex based on the high ratio of its apparent mass to its monomeric mass (R_app_ = 12.8), and it was located in a high confidence cluster with 4 other proteins (two tRNA synthetases (LYSYL-tRNA SYNTHETASE 1 (LYSRS), ISOLEUCINE-tRNA LIGASE (ILERS)), a ribosomal subunit **(**40S RIBOSOMAL PROTEIN S8–1 (RPS8A)), tubulin (β-6 TUBULIN (TUBB6) (Fig. 6*A*). In a previous publication AT2G40660 was identified as a likely protein complex subunit with an R_app_ of ∼12 that coeluted with several tRNA ligases including GLUTAMINYL-tRNA SYNTHETASE and ISOLEUCINE-tRNA SYNTHETASE (13).

Interestingly protein databases searches with AT2G40660 detected a region of high amino acid sequence conservation with human Aminoacyl tRNA synthase complex-interacting multifunctional protein 1 (AIMP1) and yeast tRNA-aminoacylation cofactor ARC1 (ARC1p), two proteins that form a cytosolic complex with multiple tRNA ligases (75–77). The region spanning amino acids 227 to 381 of AT2G40660 had the greatest amino acid similarity with AIMP1. The protein was ∼28 and 30% identical with the putative yeast and human orthologs, but the similarities were about 62 and 75%, respectively (supplemental Fig. S6). The primary function of tRNA synthetases occurs in the nucleus and is to charge tRNAs with the appropriate amino acid (78). A subset of tRNA ligases form a heteromeric complex with AIMP1/ARC1p in the cytosol as part of a signaling function independent of tRNA aminoacylation (79–81). For example, the human AIMP1 protein is a core subunit of the multi-tRNA synthetase complex (82) that is involved in glucose homeostasis (83) and inflammatory cytokine activity (84). We will refer to AT2G40660 as AIMP1-like (AIMP1L) for the remainder this article.

SEC-MS profiling was performed on wild-type and homozygous knockout line *SALK-*220E08 that contains a T-DNA in the second exon and is predicted to generate a strong loss of function allele. This study was conducted using a Thermo Q Exactive High Field mass spectrometer that had improved sensitivity compared with the AB Sciex 5600. As expected AIMP1L had a single peak and an apparent mass of ∼540 kDa in the wild-type control replicates but was not identified in the mutant (Fig. 6*B*, Top-left). We first focused on predicted AIMP1L-interactors in cluster number 64. ISOLEUCINE-tRNA LIGASE (IIERS) had an M_app_ of 541 kDa in the wild type. Unexpectedly, in *aimp1l* ILERS had an increased apparent mass of 683 kDa, a subtle ∼1 fraction shift compared with the corresponding wild-type control (Fig. 6*B*, bottom-left). A similar pattern was observed for RPS8A, which was shifted to a higher apparent mass in the mutant (Fig. 6*B*, Top-right). Glutamyl-tRNA ligase (GluRS) was not reproducibly detected in the original clustering experiment, but it did coelute in a previous profiling publication (13). In this *aimp1l* profiling experiment it displayed a pattern like ILERS and RPS8A (Fig. 6*C*). These subtle differences were not because of random fluctuations in tRNA ligase elution profiles because five other tRNA ligases that did not cluster with AIMP1L had elution profiles that were nearly identical in the wild-type and *aimp1l* (Fig. 6*D*). In the absence of AIMP1L, a subset of tRNA-ligases may assemble into distinct larger complexes that are independent of AIMP1L function. Alternatively, several AIMP1L complex subunits may dynamically rearrange among multiple complexes, and in the absence of AIMP1L, they preferentially interact with other large protein/protein complexes.

The results with LysRS and TUBB6 were less clear. Both were predicted to interact with AIM1P but in our validation experiments neither proteins clearly coeluted with AIMP1. LysRS had an M_app_ of ∼270 kDa and TUBB6 had an M_app_ of ∼110 kDa (Fig. 6*B*). However, LysRS and to a lesser degree TUBB6 displayed evidence for multiple peaks and a tendency toward a reduced oligomerization state in *aimp1l* compared with the wild type. Alanine tRNA ligase (AlaRS) was not predicted to be a member of the AIMP1L-complex based on our clustering analysis. However, in this both *aimp1l* replicates there was a slight shift of AlaRS to a smaller apparent mass and a clear secondary peak centered at ∼150 kDa (Fig. 6*C*). Perhaps LysRS and AlaRS complexes are indirectly affected by the removal of AIMP1L.

##### Validation of the Protein Complex Predictions: Coimmunoprecipitation

**Fig. 7.**
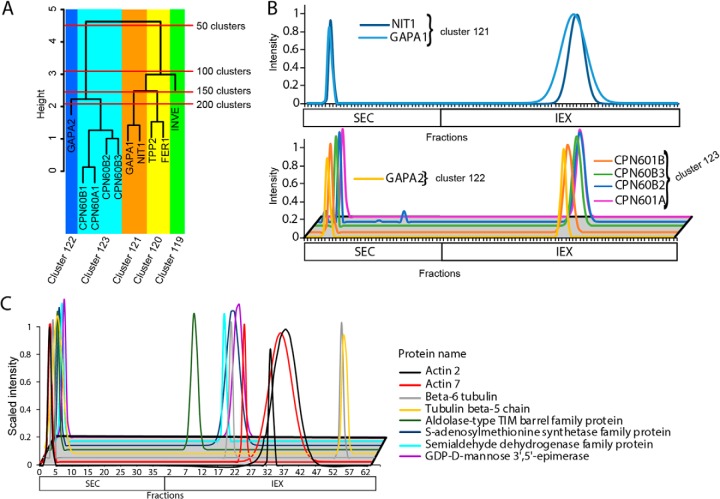
**Coimmunoprecipitation experiments to characterize the chloroplast_200_ and cytosol_300_ protein complex predictions.** *A*, The expanded dendrogram that contains Nitrilase 1 (AT3G44310), CPN60B1 (AT1G55490) and CPN60B2 (AT3G13470) that have been shown to interact by CoIP analyses. The *y* axis indicates the tree height and the red lines show were the tree is split when cut at 50 cluster increments. The dendrogram is color coded to show the complexes that were predicted when the tree was cut at 200 clusters. *B*, Nitrilase was predicted to form a complex with CPN60B2 and CPN60B1 by CoIP analysis in a previously published manuscript (21). The top panel shows the SEC and IEX elution profiles for cluster 121 that contains two proteins, NIT1 and GAPA1 (AT3G26650). The two known interactors, CPN60B1 and CPN60B2 were in cluster 123 that is close to cluster 121 containing NIT1. The lower panel shows the elution profiles for cluster 122 that falls near the NIT1 cluster and cluster 123 that contains the known interactors. *C*, CoIP with an ACTIN antibody identified multiple putative interactors that coeluted with actin in the SEC experiments (left fractions), but had distinct profiles on the IEX column (right fractions).

Another approach to validate protein complex predictions is CoIP-MS. Antibody-based purification should identify the same stable protein complexes that we detect here; however, this is unlikely to be true in all cases as different antibodies to the same protein can identify widely varying sets of interacting proteins (85). In a recent publication, Aryal *et al.*, 2017 performed CoIP analysis to identify a novel chloroplast-localized complex that contained NITRILASE1 (NIT1), CHAPERONIN 60 SUBUNIT BETA 2 (CPN60B2) and CHAPERONIN 60 SUBUNIT BETA 1 (CPN60B1). When the chloroplast_200_ prediction was queried, NIT1 (cluster 121), was found very close to CPN60B1 and CPN60B2 in cluster 123 (Fig. 7*A*). The profiles indicate NIT1, CPN60B have nearly identical peaks in SEC but, there is a slight fraction shift in the IEX separation that is driving NIT1 and the CPN proteins into slightly different clusters (Fig. 7*B*). This shows the utility of searching nearby clusters for putative interactors.

Additional CoIP experiments revealed potential artifacts likely caused by protein complex disassembly during separation on the IEX column. CoIP experiments were performed in triplicate using antibodies specific to ACTIN and GFP (to purify YFP-tagged Glyceraldehyde-3-phosphate-dehydrogenase (GAPC)) (43) and a no antibody control. Antibody purified proteins were accepted if they were absent in the negative control and detected in at least two of the three test case pull downs. Proteins that were detected in the pull downs and included in our clustering dataset were analyzed further. The actin CoIP identified included two actin isoforms and 10 additional proteins (supplemental Fig. S7*A*). CoIP of YFP:GAPC identified the target protein and the ACTIN 2 and ACTIN 7 isoforms. The actin and GAPC complex was also identified in the actin CoIP. There was considerable coelution on the SEC column of actin, GAPC, and many of the additional actin-interacting proteins identified by CoIP. For example, both ACTIN isoforms and six additional proteins coeluted at ∼950 kDa (Fig. 7*C*). GAPC and the actin isoforms also coeluted on the SEC column (supplemental Fig. S7*B*). However, there was very little coelution on the IEX column (Fig. 7C, supplemental Fig. S7*B*). We suspect that these actin-containing complexes are relatively unstable, and perhaps the combination of the TRIS-buffer exchange and high salt elution that was associated with the IEX separation caused artifactual disassembly of subsets of protein complexes. Clusters containing this type of false negative would have a relatively small number of proteins in the cluster, but the individual proteins would have a large apparent mass.

## DISCUSSION

Protein complexes integrate metabolism, transport, and signal transduction to enable complex behaviors (9, 10, 86–90). Consequently, large scale datasets that relate to protein oligomerization are highly desired (14, 22–24, 29, 30). Protein oligomerization is also dynamic: their binding-partners, assembly status, and localization change over time. Open-ended proteomic analyses of endogenous protein complexes are powerful because they provide information on subcellular partitioning (21) or how protein complexes rearrange in response to a signal (31). Obtaining large-scale datasets is challenging. A single cell type expresses over ∼10,000 proteins and based on previous protein profiling studies oligomerization is widespread (14, 18, 20, 35). In Arabidopsis leaves more than 1/3 of all proteins are predicted to oligomerize (13, 21, 32). Here we conducted orthogonal separations of protein mixtures by size and charge to reduce the confounding effect of chance coelution and developed a robust label-free proteomic profiling and data analysis pipeline to make hundreds of protein complex composition predictions.

### 

#### 

##### Creation and Partial Validation of a Protein Correlation Profiling Method for Protein Complex Prediction

Our “guilt by association” method is based on the expected coelution of subunits of stable protein complexes. The parallel size- and charge-based separations generated highly reproducible elution profiles with peaks distributed widely across all column fractions (Fig. 3*A*). Although there is coverage cost with concatenation of the SEC and IEX profile data, it decreased noise and generated dendrograms with an increased resolving power (Fig. 3*B*). Orthogonal separations enabled us to provide a highly useful dataset on predicted protein complex compositions in leaf cells.

Validation experiments showed that many clusters were highly enriched for subunits of known protein complexes (Figs. 3–5). Subunits of the 20S proteasome core particle and the RuBisCO complex (at two different assembly states, clusters 109 and 172 in the chloroplast dendrogram) were predicted with near perfect accuracy. Unannotated proteases with high sequence similarity to proteasome subunits were assigned as proteasome-associated proteins based on this analysis (Fig. 5). We do not claim that the predictions are perfect. The chloroplast interacting protein pair Nitrilase and CPN-family chaperones were in proximity in the chloroplast dendrogram, but not in the same cluster. For this reason, data users who are testing for candidate interactors of a protein of interest are referred to supplemental Table S3, which provides protein groupings at a range of cluster numbers. There are also many instances of false positives because of chance coelution and false negatives because of inadequate protein coverage or noise in the profile data (Fig. 4*D*). Complex instability during high salt elution from the IEX likely disrupted actin-containing complexes and may have revealed the relative instability of GRF1, GRF6, and GRF9 subunits. Therefore, metrics for the reliability and type of each cluster were provided for data users in supplemental Table S2, column M).

##### Discovery of a Novel tRNA Ligase Clustering Complex

Our validation studies using a *AIMP1L* mutant identifies true- and false-positive subunits of a novel aminoacyl tRNA synthetase containing complex. AIMP1L has weak homology with a vertebrate ARS complex subunit and was clustered with two class I tRNA ligases, the ribosomal protein RPS8A, and TUBB6. Removal of AIMP1L caused unexpected behaviors of predicted subunits: RPS8A, ILERS, and GLURS (a tRNA ligase that was reproducibly detected in the profiles *aimp1* with the Thermo QE but not in the clustering analysis dataset) had subtle increases in apparent mass in the mutant. The predicted interactors LYSRS and TUBB6 coeluted in the SEC column in the clustering dataset, but did not coelute in the mutant profiling experiment, suggesting they are false positives or more labile subunits. However, the oligomerization states of LYSRS and ALARS (another tRNA ligase that was reproducibly detected in the profiles *aimp1* with the Thermo QE) were altered in aimp1l (Fig. 6*B* and 6*C*). These tRNA ligases may be indirectly influenced by loss of AIMP1L and physically interact with proteins that have altered abundance or protein binding activities in *aimp1l*. This altered elution pattern in *aimp1l* was not observed with 5 other tRNA ligases that did not coelute with AIMP1L (Fig. 6*D*). The data are pointing to a broad AIMP1L-dependent protein interaction network involving many proteins protein translation. Although the *aimp1l* plant has no obvious whole-plant phenotype, this profiling analysis is a new type of phenotyping tool that can be used to develop hypotheses about gene function.

##### Useful Predictions of Self-interaction

Homo-oligomerization is a common method of enzyme regulation (3, 91) and has a strong influence on the evolution and connectivity of protein interaction networks (92). The combined use of our profiling clustering result and experimentally determined apparent masses allowed us to identify 75 cytosolic and 69 chloroplast localized proteins that are predicted to either form higher order homooligomers or assemble into complexes with a high subunit stoichiometry. This list of predicted self-interactors is riddled with proteins that have previously been shown to form homomers in nonplant species. Some proteins had an extremely high R_app_. For example, the PHOSPHOFRUCTOKINASE7 (PFK7) had an R_app_ of 18. The vertebrate PFK-L ortholog has a very high degree of polymerization, and forms filaments at the ∼100 nm spatial scale. The oligomerization of PFK7 likely has a structural importance in addition to its enzymatic function. The homomer classification also flagged NAP1 as a cytosolic homo-hexamer that may control the flux of newly synthesized histones into the nucleus. Glucosinolate production is an important form of plant chemical defense against herbivory. Based on the ability of PYK10-binding protein 1 (PYKBP1) to sediment the glucosinolate hydrolysis activity of PYK10 *in vitro*, PYK10BP1 was hypothesized to oligomerize (93). In our analysis, PYK10BP1 fell into its own cluster and had an extremely high R_app_ value of ∼16. Our data predicts that PYK10BP1 exists as a stable 16 subunit homo-oligomer under normal growth conditions. Perhaps in response to stress-dependent signal, PYK10BP1 clusters and activates PYK10. Our data also have relevance the biology of the dehydrin/COR proteins that have a known importance in plant abiotic stress response but unclear modes of action. COR family proteins have long been known to form complexes (94), and COR47 can homodimerize (95). Our data show that under nonstressed laboratory conditions the cytosolic pool of COR47 exists as a higher order oligomer (R_app_ ∼ 7). Perhaps the oligomerization state and/or binding partners of COR47 change in response to environmental stress. These selected examples were chosen to illustrate how this dataset can be used to better understand the evolution and importance of self-interaction in a wide array of physiological contexts.

##### Conclusions and Future Perspectives

Here we predict the composition of hundreds of novel protein complexes from Arabidopsis leaves. The endogenous protein correlation profiling method requires no gene cloning or tagging and can be applied to any organism with an accurate proteome. The response of putative AIMP1L-containing protein complexes to subunit removal was analyzed (Fig. 6*B* and 6*C*). This demonstrates the utility of this method to analyze the dynamics of systems of protein complexes in response to mutation, changing environmental conditions, or developmental programs. We hope that these protein complex predictions will be used by the research community to test hypotheses and provide a more complete assessment of the reliability of the dataset. Certainly, there is room for improvement. The IEX separation needs to be optimized to eliminate the buffer exchange, and better coverage will come from the continued use of the Thermo QE instrument. Additional orthogonal separations and separations done in series will help to reduce the primary technical challenge of chance coelution and occurrences of false positives. Efficient cell fractionation and the analysis of organelles will also decrease sample complexity and increase the coverage and accuracy of protein complex predictions. Our mass spec data, data filtering scripts (https://github.com/dlchenstat/ProteinComplexPredict), and final results (supplemental Tables S1–S3) are all publicly available, with the hope that these data and this method gain wide use to analyze the systems-level behaviors of endogenous protein complexes.

## DATA AVAILABILITY

The Gaussian fitting code described in McBride et. al., 2017 and deposited at (https://github.com/dlchenstat/Gaussian-fitting). The code used to generate the clustering analysis was made publicly available at (https://github.com/dlchenstat/ProteinComplexPredict). The raw mass spectrometry data was made accessible at JPOST (https://jpostdb.org/) (Identifier: PXD012601). The mass spectra are available at Protein Prospector (Search key: pqxnntlrpn, oumgx2d9bo) (http://prospector.ucsf.edu/prospector/).

## Supplementary Material

supplemental Table S2

Supplemental figures

Supplemental Table 2: Protein complex predictions

Supplemental Table 3: Clustering results using the combined cytosol and chloroplast protein profile data

Supplemental table 4: Purity of protein predictions using the combined cytosol and chloroplast protein profile data

SupplementalTable1: Peptide and Protein Abundances

SupplementalTable1: Peptide and Protein Abundances

SupplementalTable1: Peptide and Protein Abundances

Supplemental table 5

## Footnotes

^1^ The abbreviations used are:

CoIP-MS: coimmunoprecipitation-mass spectrometry
IEX: ion exchange chromatography
M_app_: apparent mass measured by size exclusion chromatography
M_calc_: calculated mass of a predicted protein complex based on the proteins in cluster
M_mono_: calculated monomer mass for a protein
P200: 200k × *g* pellet containing the microsomal fraction
R_app_: ratio of the apparent mass to the calculated mass of the monomer
S200: soluble proteins after differential centrifugation
SDS-PAGE: sodium dodecyl sulfate polyacrylamide gel electrophoresis
SEC: size exclusion chromatography
SEC+IEX: concatenated SEC and IEX protein elution profiles
TAIR: the Arabidopsis information resource.
