# Supplementary material for: A Label-free Mass Spectrometry Method to Predict Endogenous Protein Complex Composition
Source: Mol Cell Proteomics. 2019 Jun 11;18(8):1588–606. doi: 10.1074/mcp.RA119.001400 (PMC6683005; doi:10.1074/mcp.RA119.001400)
Supplement: supplemental Table S2 [file RA119.001400_index.html]

Supplement to A Label-Free Mass Spectrometry Method to Predict Endogenous Protein Complex Composition | Molecular & Cellular Proteomics

## Supplemental Data

- Supplemental figures - Supplemental figures
- Supplemental Table 2: Protein complex predictions - Protein complex predictions
- Supplemental Table 3: Clustering results using the combined cytosol and chloroplast protein profile data - Clustering results using the combined cytosol and chloroplast protein profile data
- Supplemental table 4: Purity of protein predictions using the combined cytosol and chloroplast protein profile data - Purity of protein predictions using the combined cytosol and chloroplast protein profile data
- SupplementalTable1: Peptide and Protein Abundances - SupplementalTable1: Peptide and Protein Abundances
- SupplementalTable1: Peptide and Protein Abundances - SupplementalTable1: Peptide and Protein Abundances
- SupplementalTable1: Peptide and Protein Abundances - SupplementalTable1: Peptide and Protein Abundances
- Supplemental table 5 - Cluster IDs of known protein interaction pairs in Biogrid that were present in the cytosol clustering analysis.
