# Supplementary material for: A Label-free Mass Spectrometry Method to Predict Endogenous Protein Complex Composition
Source: Mol Cell Proteomics. 2019 Jun 11;18(8):1588–606. doi: 10.1074/mcp.RA119.001400 (PMC6683005; doi:10.1074/mcp.RA119.001400)
Supplement: Supplemental figures [file 143826_2_supp_343612_psnpcl.pdf]

**Supplemental tables:**

Supplemental table 1: Raw protein and peptide abundance data

Supplemental table 2: Protein complex predictions

Supplemental table 3: Clustering results using the combined cytosol and chloroplast protein profile data

Supplemental table 4: Purity of protein predictions using the combined cytosol and chloroplast protein profile data

Supplemental table 5: Cluster IDs of known protein interaction pairs in Biogrid that were present in the cytosol clustering analysis.

**Supplemental figures:**

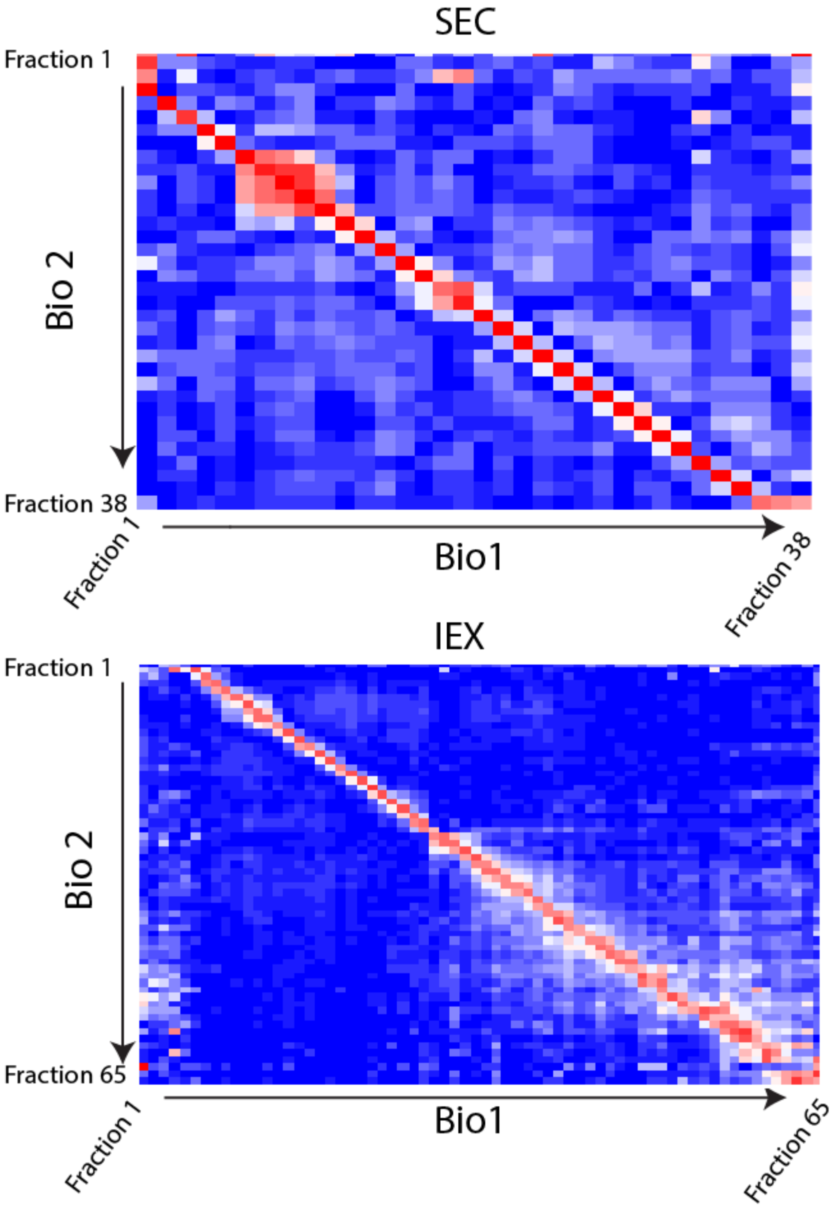


Supplemental Figure 1: Pearson correlation coefficient (PCC) matrices among all column fractions for two biological replicates. PCCs were calculated for the SEC (upper) and IEX (lower) separations using raw protein quantification values normalized to 1. The red indicates maximal PCC values and blue the minimum. Identical column fractions fall on the diagonal.

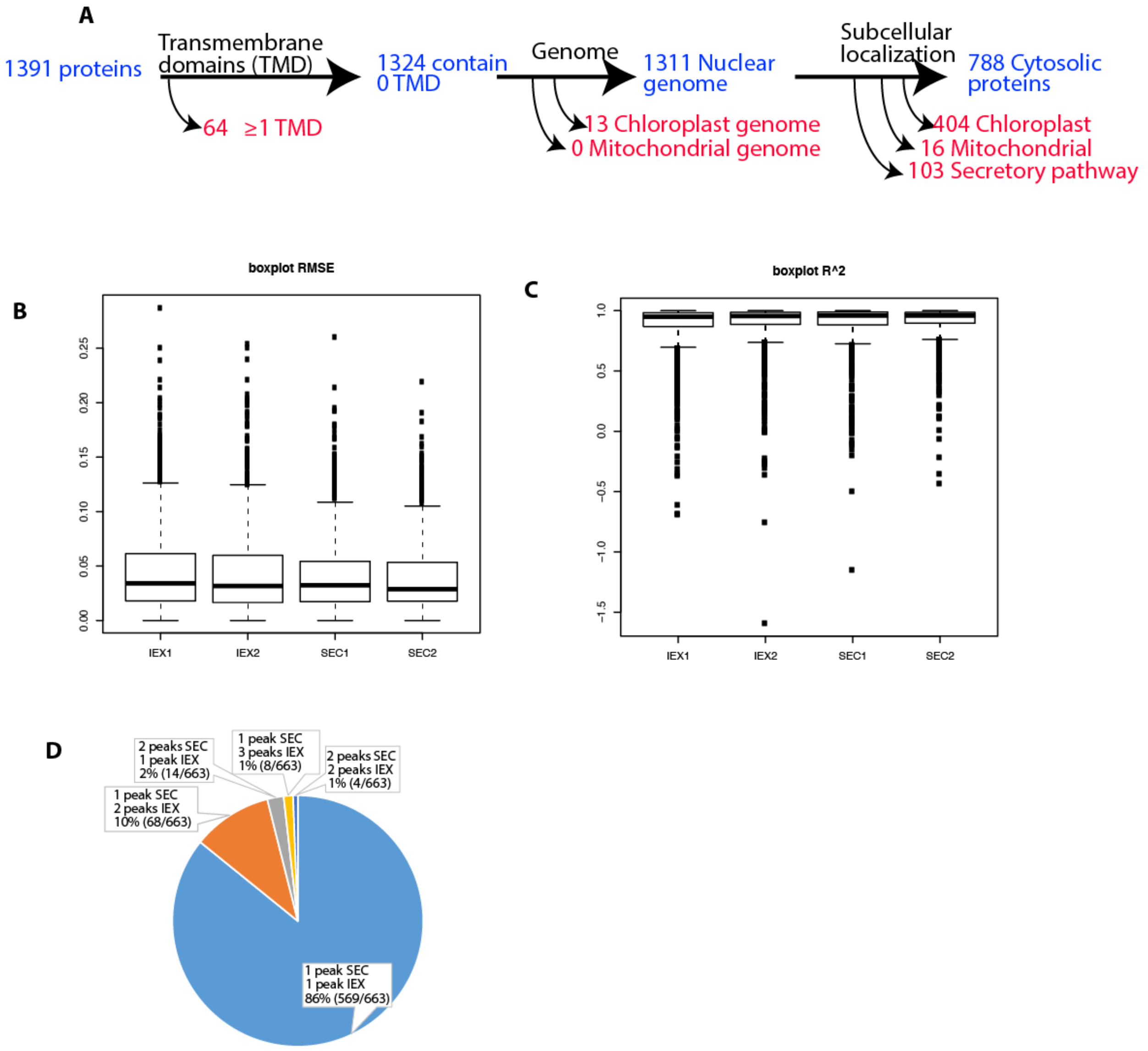


Supplemental Figure 2: Peak identification of reproducible cytosolic proteins

(A) Cytosolic proteins were predicted by filtering proteins that contained a transmembrane domain predicted by (THMM), were encoded in the nuclear genome, and were predicted to localize to the cytosol by TargetP.

(B) A boxplot of the Root Mean Squared Error (RMSE), which measures the average fitting error in the Gaussian peaks for both biological replicates for SEC and IEX.

(C) A boxplot for the R-square is the square of the correlation between the observed values and the fitted Gaussian peak values.

(D) The pie chart shows the number and percentage of proteins that contained a single or multiple reproducible peaks in SEC or IEX

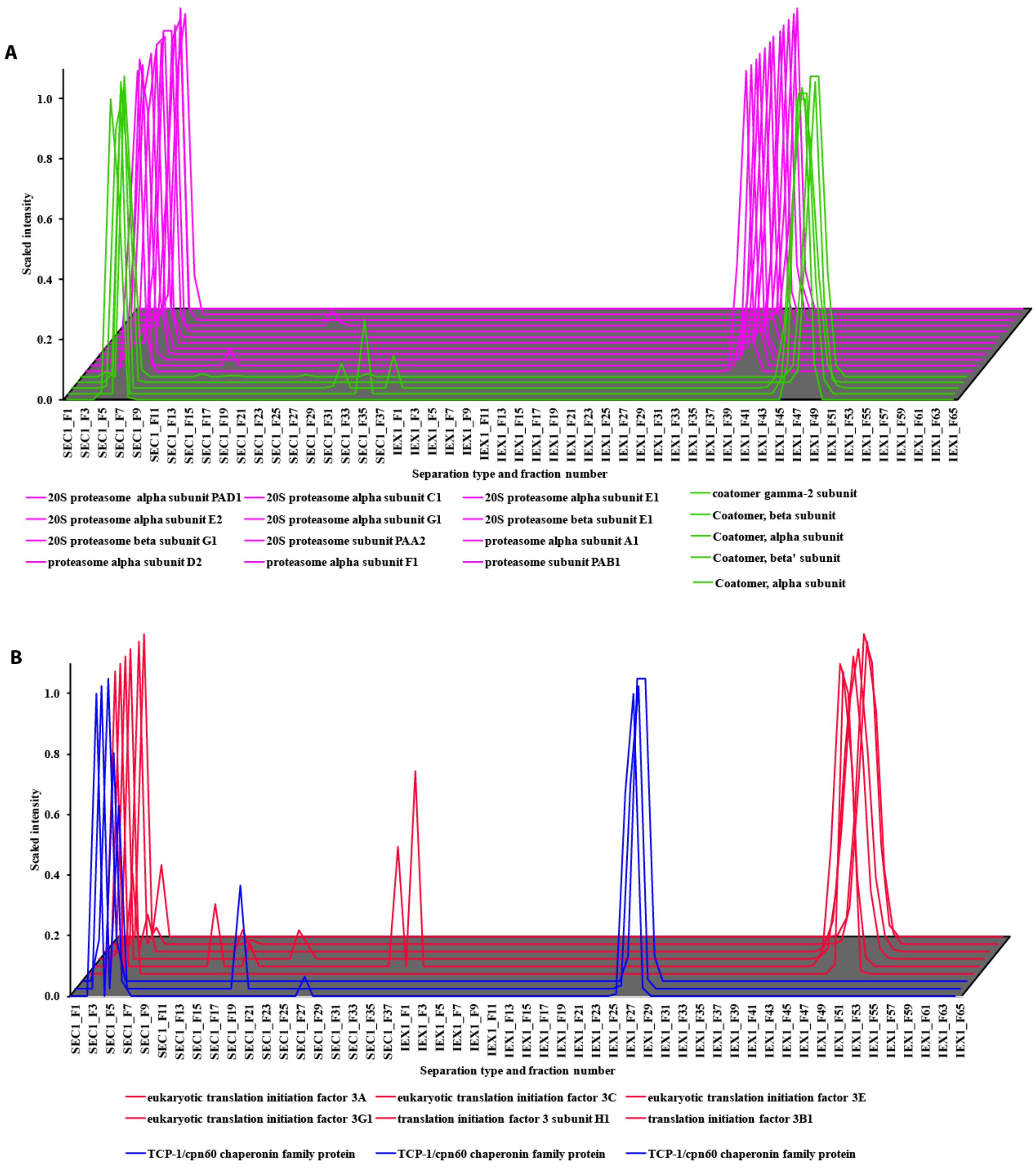
A
Scaled intensity
0.0
0.2
0.4
0.6
0.8
1.0
SEC1_F1 SEC1_F3 SEC1_F5 SEC1_F7 SEC1_F9 SEC1_F11 SEC1_F13 SEC1_F15 SEC1_F17 SEC1_F19 SEC1_F21 SEC1_F23 SEC1_F25 SEC1_F27 SEC1_F29 SEC1_F31 SEC1_F33 SEC1_F35 SEC1_F37 IEX1_F1 IEX1_F3 IEX1_F5 IEX1_F7 IEX1_F9 IEX1_F11 IEX1_F13 IEX1_F15 IEX1_F17 IEX1_F19 IEX1_F21 IEX1_F23 IEX1_F25 IEX1_F27 IEX1_F29 IEX1_F31 IEX1_F33 IEX1_F35 IEX1_F37 IEX1_F39 IEX1_F41 IEX1_F43 IEX1_F45 IEX1_F47 IEX1_F49 IEX1_F51 IEX1_F53 IEX1_F55 IEX1_F57 IEX1_F59 IEX1_F61 IEX1_F63 IEX1_F65
Separation type and fraction number
20S proteasome alpha subunit PAD1
20S proteasome alpha subunit C1
20S proteasome alpha subunit E1
20S proteasome alpha subunit E2
20S proteasome alpha subunit G1
20S proteasome beta subunit E1
20S proteasome beta subunit G1
20S proteasome subunit PAA2
proteasome alpha subunit A1
proteasome alpha subunit D2
proteasome alpha subunit F1
proteasome subunit PAB1
coatomer gamma-2 subunit
Coatomer, beta subunit
Coatomer, alpha subunit
Coatomer, beta' subunit
Coatomer, alpha subunit
B
Scaled intensity
0.0
0.2
0.4
0.6
0.8
1.0
SEC1_F1 SEC1_F3 SEC1_F5 SEC1_F7 SEC1_F9 SEC1_F11 SEC1_F13 SEC1_F15 SEC1_F17 SEC1_F19 SEC1_F21 SEC1_F23 SEC1_F25 SEC1_F27 SEC1_F29 SEC1_F31 SEC1_F33 SEC1_F35 SEC1_F37 IEX1_F1 IEX1_F3 IEX1_F5 IEX1_F7 IEX1_F9 IEX1_F11 IEX1_F13 IEX1_F15 IEX1_F17 IEX1_F19 IEX1_F21 IEX1_F23 IEX1_F25 IEX1_F27 IEX1_F29 IEX1_F31 IEX1_F33 IEX1_F35 IEX1_F37 IEX1_F39 IEX1_F41 IEX1_F43 IEX1_F45 IEX1_F47 IEX1_F49 IEX1_F51 IEX1_F53 IEX1_F55 IEX1_F57 IEX1_F59 IEX1_F61 IEX1_F63 IEX1_F65
Separation type and fraction number
eukaryotic translation initiation factor 3A
eukaryotic translation initiation factor 3C
eukaryotic translation initiation factor 3E
eukaryotic translation initiation factor 3G1
translation initiation factor 3 subunit H1
translation initiation factor 3B1
TCP-1/cpn60 chaperonin family protein
TCP-1/cpn60 chaperonin family protein
TCP-1/cpn60 chaperonin family protein

C

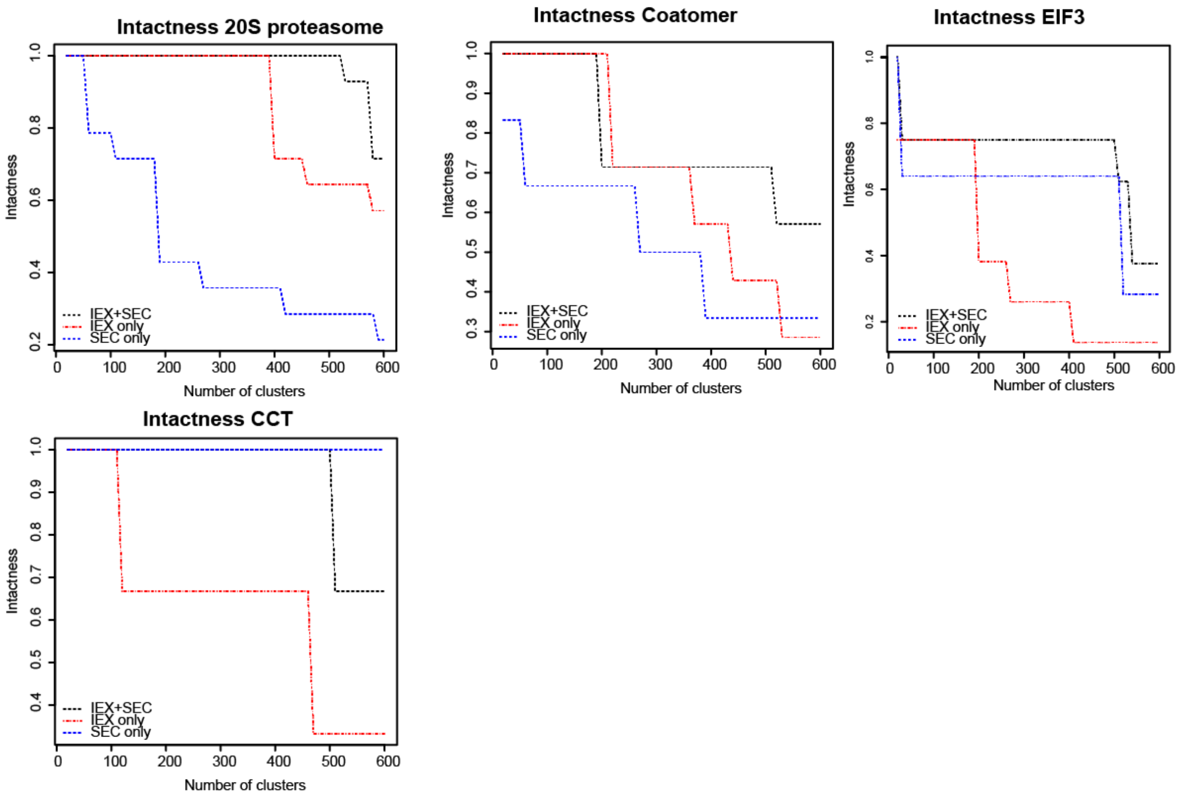
Intactness 20S proteasome
Intactness Coatomer
Intactness EIF3
Intactness CCT
Intactness
Number of clusters
IEX+SEC
IEX only
SEC only

D

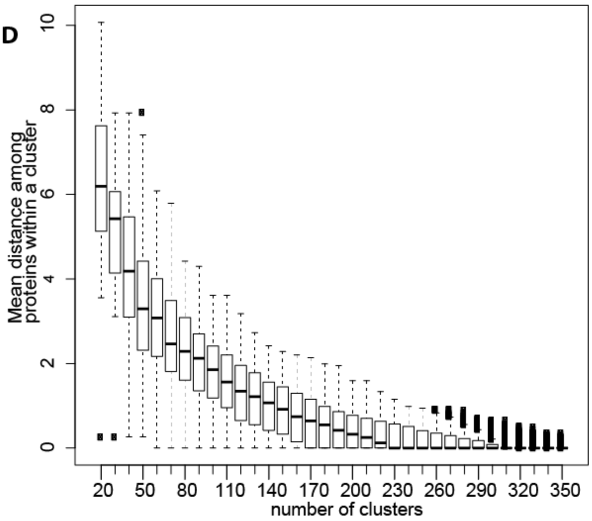
Mean distance among proteins within a cluster
number of clusters

Supplemental Figure 3: Concatenating SEC and IEX profiles increases the resolution of the protein complex prediction.

(A) The SEC and IEX fitted elution profiles for biological replicate one were plotted for the 20S proteasome and coatomer subunits. The 20S proteasome subunits are plotted in magenta and the Coatomer subunits are plotted in green. Coatamer subunit peaks are clearly separated from those of the 20S proteasome in the IEX. Locus IDs for all proteins are provided in Supplemental Table 2.

(B) The SEC and IEX fitted elution profiles for biological replicate one were plotted for the EIF3 and CCT subunits. EIF3 subunits are plotted in red and the CCT subunits are plotted in blue. Separation of the different complex subunits is obvious in the IEX. Locus IDs for all proteins are provided in Supplemental Table 2.

(C) The intactness of the known complexes was plotted for SEC only (blue lines), IEX only (red lines), and concatenated SEC and IEX profiles (black lines). Intactness is the measure of the number of subunits in a single cluster divided by the total number of subunits identified for the complex.

(D) A box plot for the distance within each cluster as a function of cluster number for the chloroplast dendrogram obtained using the combined SEC and IEX profile data. The box plot represents the first and third quartile of the data with whiskers at 1.5 of the IQR.

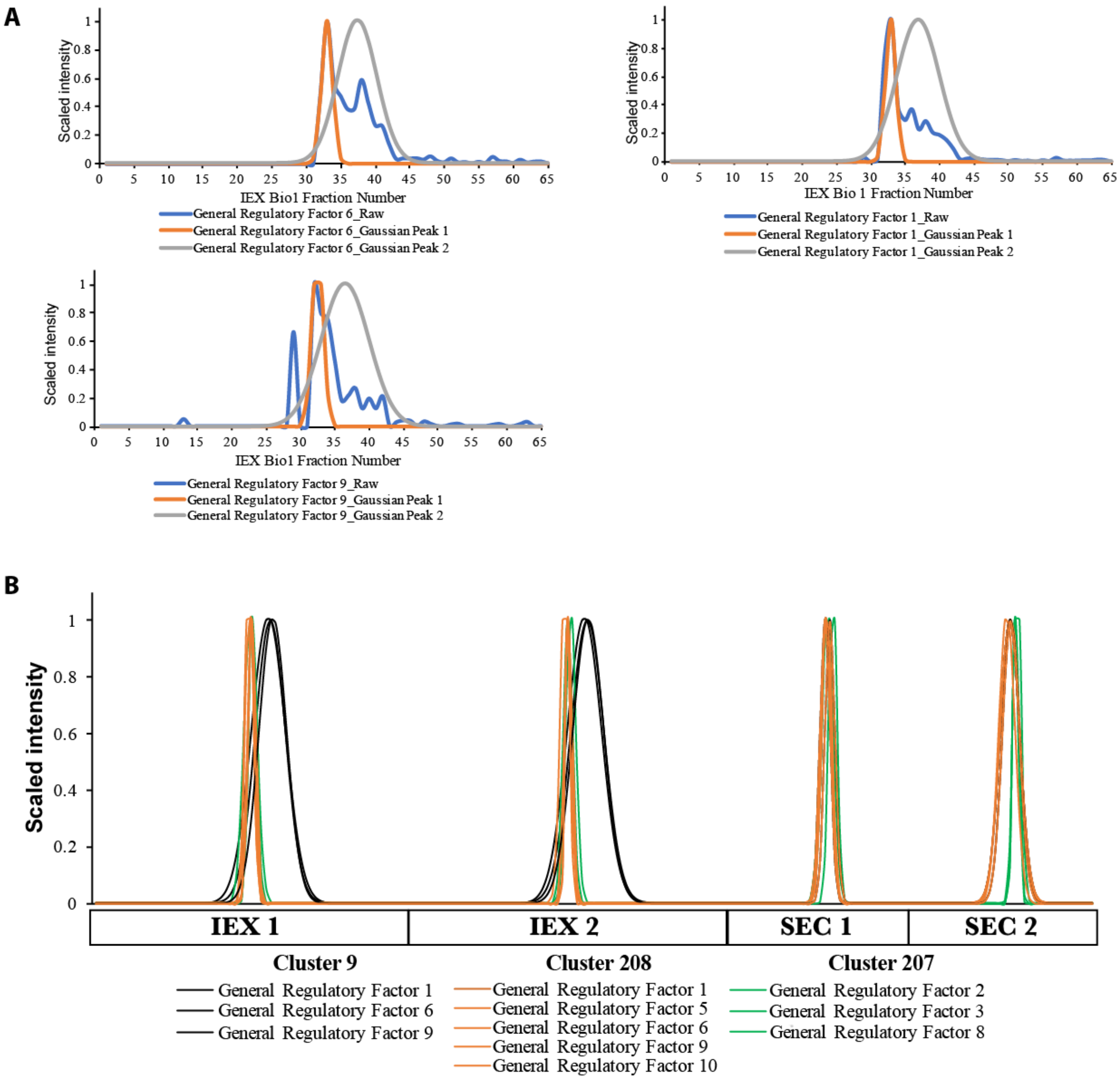


Supplemental Figure 4: A subset of GRF/14-3-3 proteins have distinct profiles on the IEX that drive their inclusion into a distinct sub-group.

(A) The raw profiles and fitted peaks for the 14-3-3/GRF proteins that had multiple peaks in the IEX (biological replicate 1 shown). Three GRF's GRF6 (AT5G10450), GRF1 (AT4G0900), and GRF9 (AT2G42590) conatined a sharp peak at ~33 and a broad beak centered near fraction 37.

(B) The fitted profiles are shown for GRFs and multiple peak GRFs that were placed into distict clusters. Cluster 9 included GRF 1, GRF 6, GRF 9. Cluster 207 contained GRF 2 (AT1G78300), GRF3 (AT5G38480), GRF 8 (AT5G65430). Cluster 208 contained GRF 1, GRF 5 (AT5G16050), GRF 6, GRF 9, and GRF 10 (AT1G22300).

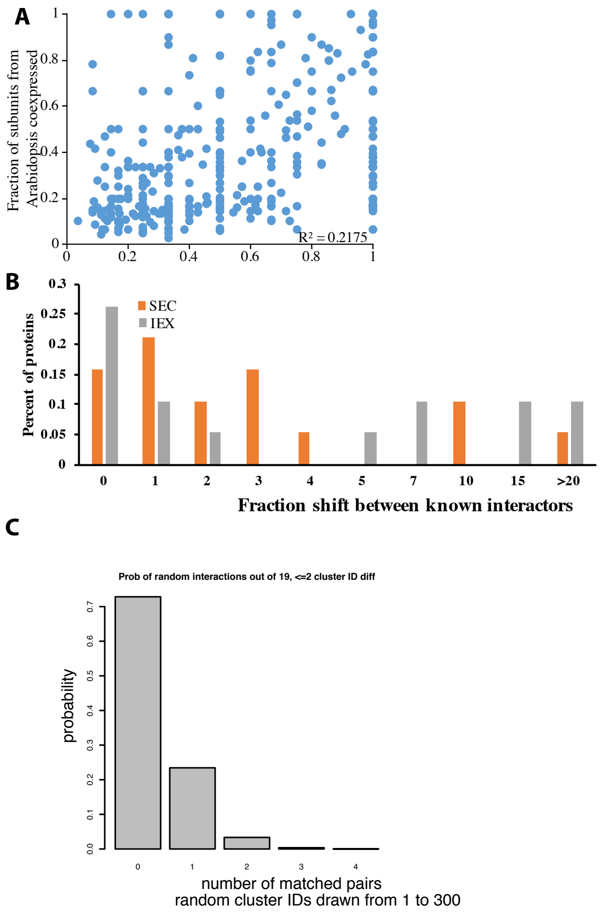


Supplemental Figure 5: Evaluation of orthogonal gene co-expression and predicted protein-protein interaction datasets as a potential datatype to refine co-elution-based protein complex predictions.

(A) Gene expression was tested to determine if it was a useful predictor of protein complex composition. The fraction of subunits that were coexpressed from known human complexes and conserved Arabidopsis complexes was plotted. Gene coexpression was taken from ATTED-II for Arabidopsis and COEXPRESSdb for human proteins.

(B) The degree of coelution of predicted protein interactors taken from Biogrid was tested. For each unique interacting pair in Biogrid, the difference in mean peak location for the SEC and IEX column separation was calculated. For example, ~15% of the pairs co-eluted in the same column fraction in the SEC separation and ~25% of the pairs co-eluted in the same column fraction in the IEX separation.

(C) Biogrid predicted interactors are enriched in our clustering prediction compared to randomly selected cluster numbers. Among the 19 protein pairs, 9 had very similar cluster IDs (cluster IDs that had a difference of less than or equal to 2, Supplemental table 5). This

level of similarity is not due to chance, because when cluster IDs were randomly drawn for the predicted interactors zero pairs were matched in over 70 percent of the simulations (n =10,000 simulations), and we never observed more than 4 matched pairs in any of the simulations.

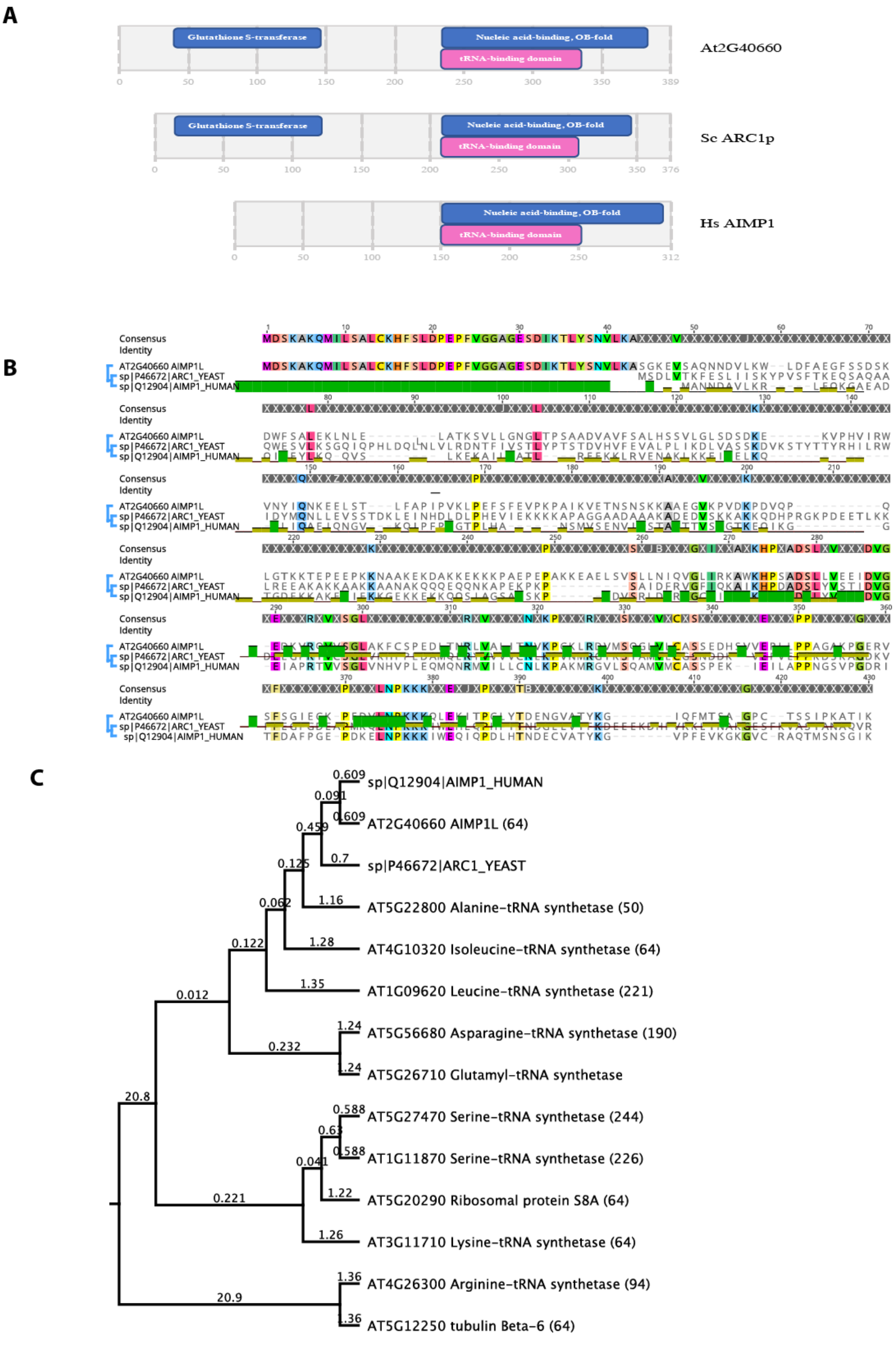
A
Glutathione S-transferase
Nucleic acid-binding, OB-fold
tRNA-binding domain
At2G40660
Sc ARC1p
Hs AIMP1
B
Consensus
Identity
AT2G40660 AIMP1L
sp|P46672|ARC1_YEAST
sp|Q12904|AIMP1_HUMAN
C
sp|Q12904|AIMP1_HUMAN
AT2G40660 AIMP1L (64)
sp|P46672|ARC1_YEAST
AT5G22800 Alanine-tRNA synthetase (50)
AT4G10320 Isoleucine-tRNA synthetase (64)
AT1G09620 Leucine-tRNA synthetase (221)
AT5G56680 Asparagine-tRNA synthetase (190)
AT5G26710 Glutamyl-tRNA synthetase
AT5G27470 Serine-tRNA synthetase (244)
AT1G11870 Serine-tRNA synthetase (226)
AT5G20290 Ribosomal protein S8A (64)
AT3G11710 Lysine-tRNA synthetase (64)
AT4G26300 Arginine-tRNA synthetase (94)
AT5G12250 tubulin Beta-6 (64)

Supplemental Figure 6: AIMP1L domain organization and sequence comparisons with homologs and tRNA synthetases.

(A) Schematic alignment of At2G40660 with yeast ARC1p (Sc ARC1p) and human AIMP1 (Hs AIMP1) shows homologous superfamily domains identified by InterProScan (http://www.ebi.ac.uk/interpro/search/sequence-search). All proteins contained Nucleic acid-binding, OB-fold (IPR012340), which contains a specific tRNA-binding domain (IPR002547). At2G40660 and Sc ARC1p possessed a Glutathione S-transferase, C-terminal domain superfamily (IPR036282).

(B) Deduced amino acid sequences of AIMP1L/At2G40660, yeast ARC1p, and human AIMP1 were aligned with MUSCLE algorism using default program parameters on Geneious Prime software (2019.0.4). Conserved amino acids were highlighted with colors unique to each individual amino acid. According to this multiple sequence alignment, the region spanning amino acids 227 to 381 (C-terminal domain) of AT2G40660 is homologous to the C-terminal domain of the yeast ARC1p, and human AIMP1.

(C) The dendrogram reflecting the relationship of a At2G40660 to yeast ARC1p and human AIMP1, and to aaRS proteins identified in this study. The distances between clades are indicated on the branches. This result was drawn with Geneious Prime software (2019.0.4).

A

| Protein Pulled down | ATG | Fasta headers | Cluster number Cyto |
|---|---|---|---|
| Actin | AT5G09810 | actin 7 | 69, 44 |
| Actin | AT3G18780 | actin 2 | 50, 4 |
| Actin | AT1G42970 | glyceraldehyde-3-phosphate dehydrogenase B subunit | 27, 16 |
| Actin | AT5G14740 | carbonic anhydrase 2 | 192 |
| Actin | AT5G02500 | heat shock cognate protein 70-1 | 55, 54 |
| Actin | AT3G14415 | Aldolase-type TIM barrel family protein | 273 |
| Actin | AT3G13920 | eukaryotic translation initiation factor 4A1 | 54 |
| Actin | AT5G28840 | GDP-D-mannose 3',5'-epimerase | 265 |
| Actin | AT3G17390 | S-adenosylmethionine synthetase family protein | 264 |
| Actin | AT5G12250 | beta-6 tubulin | 64, 53 |
| Actin | AT1G14810 | semialdehyde dehydrogenase family protein | 62 |
| Actin | AT1G20010 | tubulin beta-5 chain | 53 |
| GAPC2 | AT3G04120 | glyceraldehyde-3-phosphate dehydrogenase C subunit 1 | 66 |
| GAPC2 | AT3G18780 | actin 2 | 50, 4 |
| GAPC2 | AT5G09810 | actin 7 | 69, 44 |

B

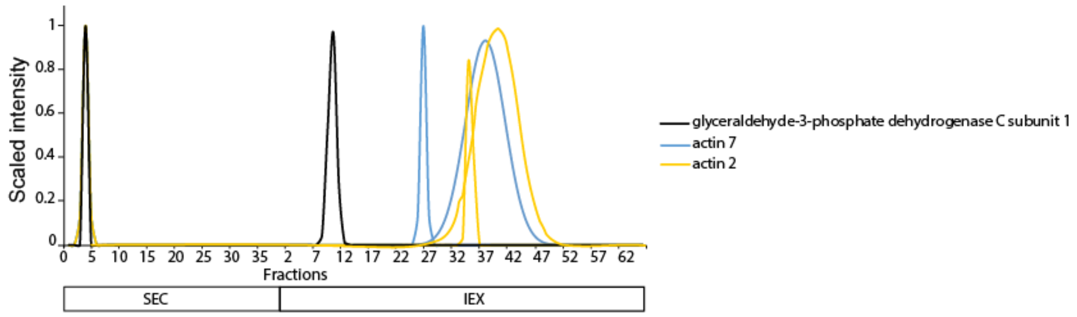


Supplemental Figure 7: Coimmunoprecipitation of actin and GAPC-binding proteins that were detected in the clustering analysis

(A) A table listing the proteins identified by CoIP and the cluster number of the identified protein.

(B) Profiles SEC and IEX profiles of the GAPC2-interactors.
